# Promoter-like epigenetic signatures in exons displaying cell type-specific splicing

**DOI:** 10.1186/s13059-015-0797-8

**Published:** 2015-10-23

**Authors:** Joao Curado, Camilla Iannone, Hagen Tilgner, Juan Valcárcel, Roderic Guigó

**Affiliations:** Centre for Genomic Regulation (CRG), The Barcelona Institute of Science and Technology, Dr. Aiguader, 88, 08003 Barcelona, Catalonia Spain; Graduate program in Areas of Basic and Applied Biology, Abel Salazar Biomedical Sciences Institute, University of Porto, 4099-003 Porto, Portugal; Universitat Pompeu Fabra, Dr. Aiguader, 88, 08003 Barcelona, Catalonia Spain; Department of Genetics, Stanford University, 300 Pasteur Dr., Stanford, CA 94305-5120 USA; Institució Catalana de Recerca i Estudis Avançats, Pg Lluis Companys 23, 08010 Barcelona, Catalonia Spain

**Keywords:** Chromatin, Splicing, Histone, Modifications, Promoters, Proximity, Transcription, Epigenetic, RNA, ENCODE

## Abstract

**Background:**

Pre-mRNA splicing occurs mainly co-transcriptionally, and both nucleosome density and histone modifications have been proposed to play a role in splice site recognition and regulation. However, the extent and mechanisms behind this interplay remain poorly understood.

**Results:**

We use transcriptomic and epigenomic data generated by the ENCODE project to investigate the association between chromatin structure and alternative splicing. We find a strong and significant positive association between H3K9ac, H3K27ac, H3K4me3, epigenetic marks characteristic of active promoters, and exon inclusion in a small but well-defined class of exons, representing approximately 4 % of all regulated exons. These exons are systematically maintained at comparatively low levels of inclusion across cell types, but their inclusion is significantly enhanced in particular cell types when in physical proximity to active promoters.

**Conclusion:**

Histone modifications and other chromatin features that activate transcription can be co-opted to participate in the regulation of the splicing of exons that are in physical proximity to promoter regions.

**Electronic supplementary material:**

The online version of this article (doi:10.1186/s13059-015-0797-8) contains supplementary material, which is available to authorized users.

## Background

Alternative pre-mRNA splicing is assumed to expand the diversity of mRNAs encoded in the genome. The prevalence of alternative splicing increases from invertebrates to vertebrates [1] and is particularly high in the immune and nervous systems, where high diversity of molecular repertoires is necessary for cell identity [2]. Whether an alternative exon is included or excluded in a mature RNA is considered a matter of combinatorial control, involving splice sites, additional binding sites, and the factors that recognize them [3]. Recent evidence suggests that chromatin organization and transcriptional dynamics may also contribute to this control. First, splicing can occur co-transcriptionally [4, 5], and this has been demonstrated to be widespread in yeast [6], fruit fly [7], and human [8, 9]. Second, some splicing factors are known to interact with modified histone tails, and intragenic histone modifications have been shown to be involved in alternative splicing decisions on individual genes [10]. Third, RNA Polymerase II elongation dynamics is known to influence exon inclusion [11, 12], which was shown to be modulated by CTCF binding [13]. Lastly, a number of independent studies demonstrated that nucleosome density correlates with exon-intron architecture genome-wide [9, 14–19], and specifically with exon inclusion levels [20, 21].

While links between chromatin and splicing have thus been established, attempts to incorporate chromatin information on quantitative models predictive of cell type-specific exon inclusion levels have met so far with moderate success [22], and the extent to which cell type-specific chromatin organization contributes to cell type-specific splicing patterns remain largely unknown.

Here to investigate the relationship between chromatin and splicing, we analyzed transcriptome and epigenome data generated in a number of human cell lines within the ENCODE project [23, 24]. First, we used RNASeq data to identify exons that are differentially included between cell lines. We found that a relatively small fraction of human exons (about 3 % of all internal exons in about 10 % of all investigated genes) exhibit regulated inclusion across human cell lines. These regulated exons are maintained at intermediate inclusion levels compared to all exons. Second, we used ChIPSeq data to investigate the association between the inclusion of regulated exons and histone modifications. Our results strongly suggest that there is little or no direct association between histone modifications and the inclusion levels of the majority of these exons. We identified, however, a small set of regulated exons (about 4 % of all differentially included exons) in which cell type-specific inclusion levels do appear to be directly associated to levels of canonically activating histone modifications. In contrast to most exons, the inclusion of these exons is maintained at remarkable low levels across a large variety of cell types and tissues. In addition to being enriched in histone modifications, these exons have other characteristics typical of promoter regions, but they do not correspond to sites of transcription initiation. However, they tend to lie closer to transcription initiation sites, and through chromatin looping they tend to interact with promoter regions.

Our observations are consistent with a role for promoter regions and for promoter-characteristic epigenetic signatures in the regulation of the alternative splicing of a well-defined set of exons, possibly involving the opening of chromatin and folding of chromatin loops that bring together regulated exons and promoters into close spatial distance. Histone modifications and other features that activate transcription could then be co-opted in these cases to participate also in the regulation of exon inclusion.

## Results

### R1. Alternatively included exons in pair-wise cell type comparisons

**Fig. 1:**
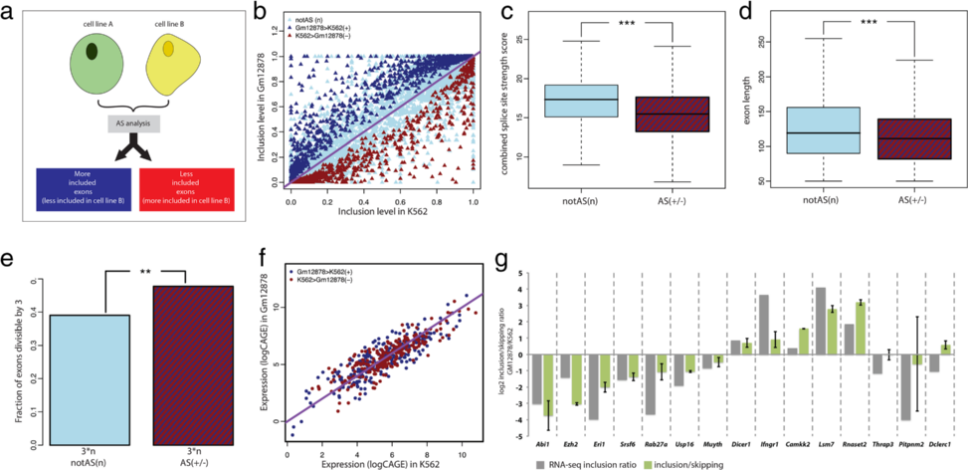
Assessment and properties of exons differentially spliced between cell lines K562 and Gm12878. **a** Classification of ‘more included’ and ‘less included’ exons. **b** Estimated inclusion ratio in the K562 cell line (x-axis) and in the Gm12878 cell line (y-axis) of exons whose inclusion is: (1) significantly higher in Gm12878 (dark blue); (2) significantly higher in the K562 cell line (dark red); (3) whose inclusion does not change significantly between the two cell lines (light blue). **c** Distribution of the sum of the strengths of 5’ and 3’ splice sites flanking exons that do not display significant differences in inclusion between the cell lines (notAS, left boxplot) and differentially included (that is, regulated) exons (AS, right boxplot). Wilcoxon rank-sum tests were calculated for the two distributions, significance levels are indicated: * (0.05 > *P* > 0.01), ** (0.01 > *P* > 0.001), *** (0.001 > *P*). **d** Exon length distribution of AS and notAS exons. **e** Fraction of AS and notAS exons, the length of which is multiple of three: only exons that were entirely coding were considered. **f** Expression of genes with exons whose inclusion is: (1) significantly higher in Gm12878 (dark blue); (2) significantly higher in the K562 cell line (dark red). X-axis: log2 (TSS-HMM value) in K562; Y-axis: log2 (TSS-HMM value) in Gm12878. **g** Experimental validation: comparison between inclusion levels of differentially regulated exons between Gm12878 and K562, calculated analyzing RNASeq ENCODE data (gray bars) or with RT-qPCR analyses of RNA extracted from K562 and Gm12878 (green bars). For RT-qPCR analysis were used primers amplifying specifically the inclusion or skipping isoform. Gray bars represent log2 ratio of inclusion level between Gm12878 and K562 calculated for each exon form RNASeq. Green bars represent log2 ratio between inclusion/skipping ratio in Gm12878 and K562, normalized to the ratio in a constitutive exon on the same transcript; error bars represent standard deviations of three independent experiments. Twelve out of 15 exons tested show consistent inclusion direction as measured by RNASeq and RT-qPCR

We used nuclear polyA+ RNASeq samples from five Tier 1 human cell lines from the ENCODE project (K562, Gm12878, Hepg2, Huvec, Helas3) [25] to identify differentially included internal exons in pair-wise comparisons of cell lines. We used a method similar to that published by Wang *et al.* [26] (Fig. 1a, Methods, Additional file 1: Figure S1). Nuclear polyA+ RNA was selected, since, in contrast to other RNA fractions, in this fraction splicing has been essentially completed [9], but it is unlikely to have undergone nonsense mediated decay (NMD), and thus, it reflects more precisely the direct outcome of splicing.

**Fig. 2:**
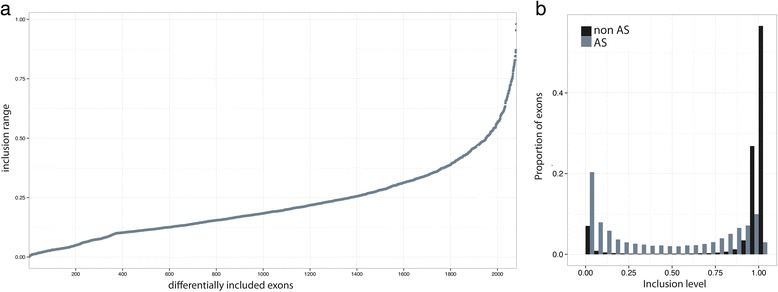
Differentially spliced exons in cell lines. **a** Inclusion range of regulated exons. The inclusion range of an exon is defined as the difference between the maximum and the minimum inclusion observed for that exon across the cell lines investigated. Exons are sorted by inclusion range. **b** Distribution of the inclusion level of regulated (AS) and non-regulated exons (notAS) across all the cell lines used

We selected 73,329 internal exons with canonical splice junctions that lay at least 600 bp away from the closely annotated Transcription Start Site (TSS) and Transcription Termination Site (TTS), and for which there were enough RNASeq reads to compute differential inclusion in at least one pairwise cell comparison (see Methods). These belong to 15,679 different genes. We used the one-sided Fisher test on the number of exclusion and inclusion reads to identify differentially included exons between each cell pair (see Methods). Exons with significant inclusion level changes were called more/less included exons depending in the direction of the change (Fig. 1a). Figure 1b–g shows the results of the comparison of Gm12878 and K562. We identified 1,688 exons regulated between these two cell lines (1,066 more included in Gm12878 and 622 more included in K562, *P* value <0.05, Fig. 1b). These differentially included exons possess known properties of alternative exons, as previously described in the literature [27, 28]: (1) they have weaker splice sites compared to exons not differentially included (Fig. 1c); (2) they tend to be shorter (Fig. 1d); and (3) when coding, their length is more often divisible by three (Fig. 1e). Moreover, (4) we did not find significant differences in the expression levels of the genes hosting differentially included exons between the two cell lines compared (Fig. 1f). In order to independently validate our alternative exon calling method, we selected a total of 15 of these exons (Fig. 1g). Using exon-junction oligonucleotides (Additional file 1: Table S1), we quantified by qPCR the ratio of inclusion/skipping isoform in Gm12878 compared to K562. This method of quantification provides an assessment of the differences in the relative inclusion of the exons regardless of possible differences in gene expression between the cell lines compared. We validated 12 out of the 15 selected cases (Fig. 1g), corresponding to a validation rate of 80 %.

We assessed whether conditions 1–4 above (Fig. 1c–f) were satisfied in each of the 10 pairwise comparisons between the five cell lines considered here, and we kept only the seven comparisons satisfying all of them (Additional file 1: Table S2). Furthermore, we retained only exons with minimum absolute change of 0.1 or two-fold in the inclusion levels between the two cell lines. In total, we obtained 1,849 more included and 2,483 less included exon comparisons in the seven cell-pairs employed. The terms ‘more included exons’ and ‘less included exons’ are arbitrary, since they depend on the direction of the comparison. We preferred to keep them separate to allow for better visualization and validation of the results presented (see below).

Because the same exon can appear in different pairwise comparisons, when pooled together, the two sets correspond to 2,081 unique exons that showed regulated inclusion levels across the human ENCODE cell lines. These correspond to about 3 % of all exons initially considered, and belong to 1,637 genes (10.44 % of all genes initially considered). Exons with regulated inclusion exhibit in general weak inclusion changes across human cell lines (Fig. 2a). The median exon inclusion range (that is, the differences between the maximum and minimum inclusion observed) is 0.20. For more than 90 % of the exons, the change is less than 0.5. Moreover, they show generally intermediate exon inclusion levels when compared with the inclusion levels of non-regulated exons (Fig. 2b).

Because gene expression levels are linked to chromatin organization [29] and can also influence splicing [3], we excluded exons from genes showing large (more than 10-fold) expression differences between cell lines. In addition, in our analysis, we used only one exon per gene, the one with the lowest *P* value. In total, we obtained 1,684 more included and 2,198 less included exon comparisons in the seven cell pairs employed corresponding to 1,921 unique exons (Additional file 1: Table S3, Additional files 2 and 3). The splicing landscape of this filtered set of regulated exons is very similar to the previous one (Fig. 2 and Additional file 1: Figure S2).

### R2. Co-occurrence of differences in histone modifications with alternative exon inclusion

For each differentially included exon in each pairwise cell comparison, we computed differential signal for nine histone marks, the insulator protein CTCF and input DNA [24, 30, 31]. We defined the ‘differential signal’ for each chromatin feature as the difference of the averaged normalized signal over the exon in the second cell type (for example, Gm12878 for the comparison K562 vs. Gm12878) and the averaged normalized signal over the exon in the first one (for example, K562 for the same comparison, see Methods). We pooled the differential exonic signal across all exons and all cell-line comparisons together to produce a single composite comparison for each monitored variable separately for ‘more included’ and ‘less included’ exons. By using differential signals on the same genomic interval, we eliminated possible biases due to intrinsic genomic features such as GC content.

**Fig. 3:**
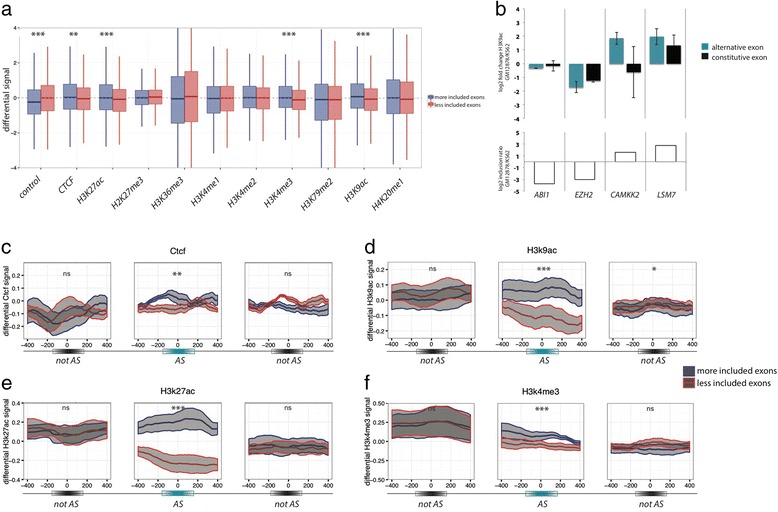
Enrichment of chromatin epigenetic marks on regulated exons. **a** Differential signals (log2, Y-axis) for ‘more included’ (blue) or ‘less included’ (red) exons from the seven cell pairs used, are represented for 11 ChIPSeq datasets corresponding to different epigenetic marks, CTCF, and input DNA (control). The boxplots correspond to the distribution of the average differential signal over the length of each regulated exon. Wilcoxon rank-sum tests with Bonferroni correction were calculated for the two distributions, significance levels are indicated: * (0.05 > *P* > 0.01), ** (0.01 > *P* > 0.001), *** (0.001 > *P*). CTCF, H3K9ac, H3K4me3, and H3K27ac have significantly higher signal in ‘more included’ than in ‘less included’ exons, while input DNA control shows the opposite trend. **b** Validation of H3K9ac enrichment over H3 by ChIP. Average and standard deviation of log2 of the fold change in H3K9ac signal over total H3 signal in regulated (alternative) and constitutive exons from four different genes. Values are from three independent replicates. White bars in the lower panel represent inclusion level ratios between the two cell lines for the regulated exons, as determined by RNASeq. A general association between exon inclusion and higher levels of H3K9 acetylation is observed. **c**–**f** Differential ChIPSeq signals (average and standard error of the mean) for CTCF, H3K9ac, H3K27ac, and H3K4me3 are represented for ‘more included’ exons (blue) and ‘less included’ exons (red) in a 800 bp-window around the middle of the regulated exon (AS) and flanking not regulated (notAS) upstream (left) and downstream (right) exons. Significance levels are indicated by * (0.05 > *P* > 0.01), ** (0.01 > *P* > 0.001), *** (0.001 > *P*), and ns (*P* > 0.05). Differential accumulation of marks is generally specific of regulated exons

Our analysis revealed enrichment of CTCF, H3K9ac, H3K27ac, and H3K4me3 levels in more included exons (*P* value <0.01; Wilcoxon signed-rank test with Bonferroni correction) (Fig. 3a). We also observed a negative association with the control signal, which consists of cross-linked and sonicated DNA. This may represent a measure of chromatin compaction, and may reflect an association between open chromatin and higher levels of exon inclusion. Importantly, the association observed between exon inclusion and input DNA is in the opposite direction than that observed for rest of the chromatin features, indicating that we are likely underestimating the strength of the associations. To validate these results, we performed ChIP-qPCR using H3K9ac antibodies and primers specific (Additional file 1: Table S4) for the target exons (and a constitutive exon of the same gene as a control) in K562 and Gm12878 cells. In all four alternative exons investigated, a clear difference in H3K9ac signal between the two cell lines was detected, positively correlating with differential exon usage. In contrast, constitutive exons on the same genes showed, in general, smaller (sometimes even opposite) differences and higher variability among replicates (Fig. 3b).

To assess whether the differential enrichment in histone modifications was local and specific of the regulated exons, or rather affected more extensive regions of the gene, we analyzed the distribution of epigenetic marks in the closest upstream and downstream exons that our method did not identify as differentially included (Methods). This defines a ‘not regulated -regulated - not regulated’ exon triplet. Differential chromatin profiles were calculated within 800 bp windows from the center of the exons. The results showed that the enrichment in chromatin signals does not extend to the flanking exons and it is, therefore, specific of the differentially included exons (Figs. 3c–f). This confirms a significant positive association between exon inclusion and local levels of H3K9ac, H3K27ac, H3K4me3, and CTCF binding.

### R3. Promoter-like histone marks and exon inclusion

**Fig. 4:**
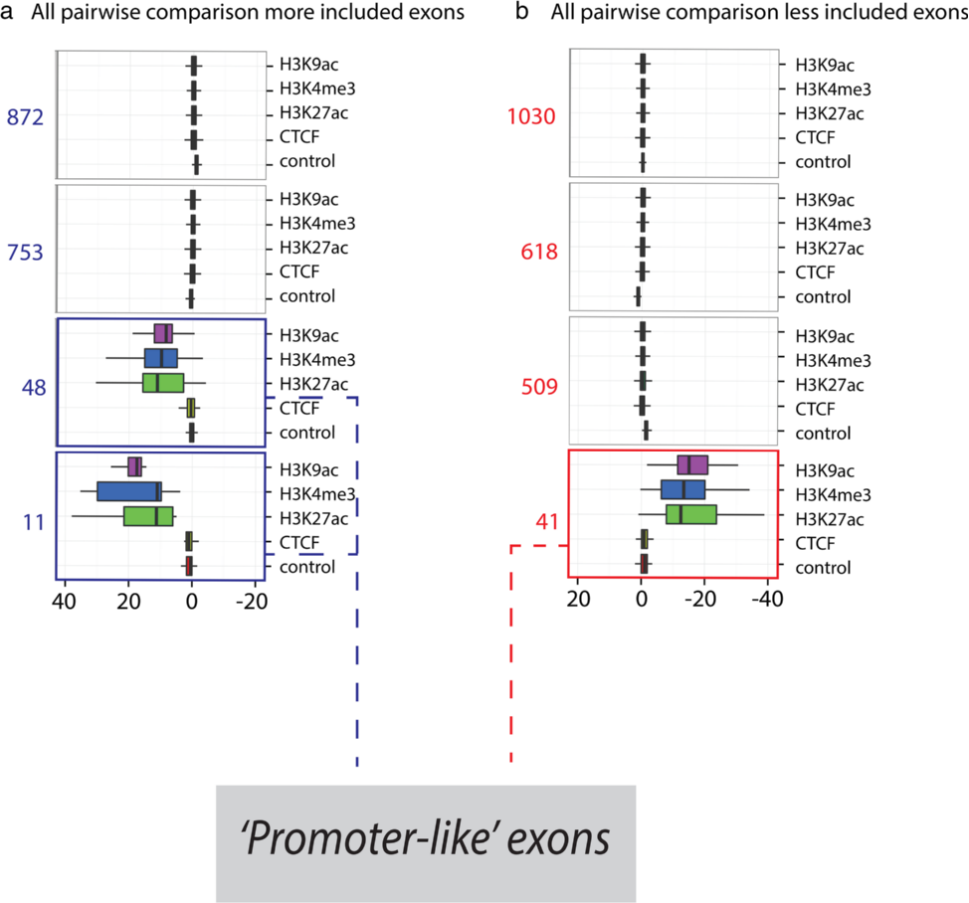
K-means clustering based on epigenetic signatures of regulated exons. **a**, **b** boxplots represent differential ChIPSeq signal for more included (**a**) and less included (**b**) exons. Each square represents one group of exons identified by K-means clustering. The number of exons present in each cluster is indicated to the left of each cluster. Three clusters of exons show strong differential signal for H3K9ac, H3K27ac, and H3K4me3 in the direction of the inclusion level change. These exons were called ‘promoter-like’ exons due to the nature of these histone modifications

While the results above do indicate a significant association between a number of histone modifications and exon inclusion, the effect is certainly weak. This could reflect a general, but weak, effect of histone modification on most differentially included exons, or alternatively, a strong effect only on a subset of them. To investigate the two alternatives, we performed k-means clustering on the sets of more and less included exons, based on the levels of the five chromatin features that we found significantly associated with differential exon inclusion. After k-means optimization, the data were partitioned in four clusters of different signal profiles and each exon was assigned to the cluster with the nearest mean (Fig. 4, Methods). As expected ‘more included’ and ‘less included’ exons generated similar but mirrored clusters.

The k-means clustering shows clearly that while the majority of differentially included exons do not show differences in the levels of the monitored histone modifications between cell pairs, a subset of 4 % of all differentially regulated exons (100 exon comparisons) exhibits co-occurrence of large differential levels of H3K9ac, H3K27ac, and H3K4me3 associated with differential exon inclusion. Surprisingly, for the clusters that exhibit a change in the differential level of histone modifications, this change is always concordant with the direction of the inclusion level change (Fig. 4, Additional file 4). This was not the case when performing clustering with the histone modifications levels in the downstream constitutive exons (Additional file 1: Figure S3).

Since these marks are known signatures of active promoters, we will refer to this subset of exons as ‘promoter-like’ exons. These 100 exon comparisons are distributed across all cell-line pairs considered here (Additional file 1: Table S5) and they correspond to a total of 70 unique exons, as some of these exons appear in multiple comparisons. Principal Component Analysis (PCA) reinforced the hypothesis that these exons have peculiar features regarding histone modifications. Indeed PCA based on the differential histone modification levels clearly separates the core set of non-‘promoter-like’ exons, the histone modifications levels of which is immune to splicing changes, from the ‘promoter-like’ exons, which spread in all directions towards the periphery of the PCA projection (Additional file 1: Figure S4).

To further validate these findings, we compared K562 and NHEK, an ENCODE Tier 2 cell line that had not been previously used in our analysis. Out of the 70 exons above, 22 are differentially included between these two cell lines. In two cases, no change in histone modification levels could be detected. In 17 of the remaining 20 (85 %), the direction of the differential inclusion is consistent with the direction of the total differential levels of H3K9ac, H3K27ac, and H3K4me3 (Additional file 1: Figure S5), while only in three cases (15 %) exon inclusion and histone modification levels change in opposite directions. With the same rationale, we compared NHEK with the other cell lines used in our analysis (Gm12878, HelaS3, Huvec, HepG2) and expanded our validation to 40 out of 47 different exons tested (Additional file 1: Table S6). Across all comparisons we obtained 73 % of correct prediction, with a median validation rate of 71 %.

### R4. Characterization of ‘promoter-like’ exons

We thus proceeded to further characterize the subset of ‘promoter-like’ exons. We merged the 100 ‘promoter-like’ exon comparisons from the more-included and less-included groups, retaining the association with the pair of cell lines where they were identified as differentially included. For each exon comparisons we then have one exon associated with two cell lines: C-higher (the cell line in which the exon is more included) and C-lower (the cell line in which it is less included). For a list of all the ‘promoter-like’ exons and the corresponding C-higher and C-lower cell lines, see Additional file 1: Table S7.

**Fig. 5:**
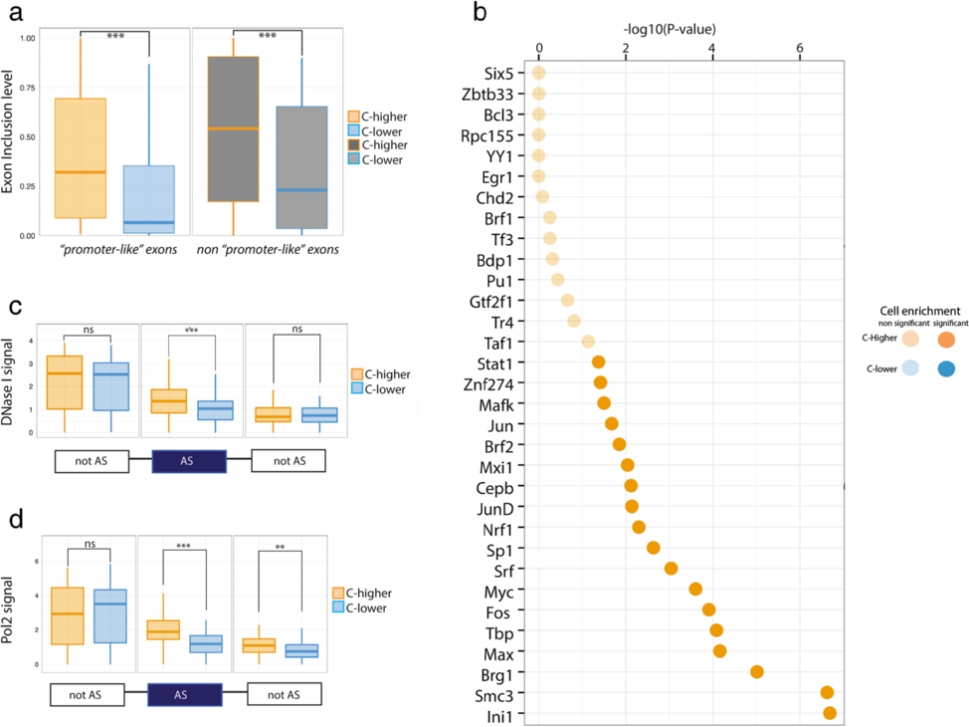
Characterization of “promoter-like” exons. **a** Exon inclusion levels in C-higher (yellow) and C-lower (blue) cell lines in “promoter-like” and non “promoter-like” exons. **b** Transcription factors binding enrichment significance in C-higher over C-lower cell lines. Bonferroni corrected –log10(p-value) of the enrichment is represented for all the transcription factors tested. All the transcription factors had higher signal in C-higher (orange). No transcription factor was found enriched on C-lower cell lines in “promoter-like” exon, thus there is no blue dot represented on the graph. Solid colors represents significant enrichments (p < 0.05) (**c-d**) DNase I sensitivity and RNA polymerase II signals in promoter-like exons in C-higher and C-lower cell lines. Signals are represented for regulated (AS) and flanking non-regulated (notAS) exons. For input relative to panel (**d**) see Additional file 1: Figure S11

Compared to the rest of regulated exons, ‘promoter-like’ exons are included at particularly low levels, with a median inclusion level of 0.32 and 0.07 in ‘C-higher’ and ‘C-lower’ cell lines, respectively (see exon inclusion distribution in Additional file 1: Figure S6), in comparison with 0.54 and 0.23 for the non-‘promoter-like’ regulated exons (Fig. 5a). To assess whether low inclusion levels are a characteristic of ‘promoter-like’ exons, or a consequence of our measurements restricted to human cell lines, we used 1,500 RNASeq samples from the GTEx project [32] to estimate exon inclusion levels of the set of regulated exons in human tissues. We found that also in human tissues, ‘promoter-like’ exons exhibit significantly lower levels of inclusion that regulated non-‘promoter-like’ exons (Additional file 1: Figure S7).

‘Promoter-like’ exons are characterized by additional promoter-associated features when compared to the rest of regulated exons. First, they are enriched in binding sites, both when considering sequence motifs (Additional file 1: Tables S8 and S9), and accumulation of Transcription Factor (TF) ChIPSeq reads. Indeed, we found that 15 out of the 32 TF analyzed have significantly more accumulation of reads in C-higher than in C-lower cell lines, while none displays the opposite trend (Fig. 5b and Additional file 1: Figure S9). Among the enriched transcription factors is Brg1, which together with Brm is one of the two ATPases of the chromatin remodeling complex SWI/SNF, and it has been shown to interact with the splicing machinery [33] (Additional file 1: Figure S9). Second, they are enriched in DNase I hypersensitive sites (DHS) in C-higher cell lines (Fig. 5c). DHS generally mark *cis*-regulatory elements and are indicative of open, more accessible chromatin. The difference in DHS signal is limited to the regulated exons and does not expand to the flanking constitutive ones (Fig. 5c). All differentially regulated exons show a similar but weaker enrichment (Additional file 1: Figure S9). Third, ‘promoter-like’ exons in C-higher also show a clear enrichment of RNA Polymerase II signal (Fig. 5d).

**Fig. 6:**
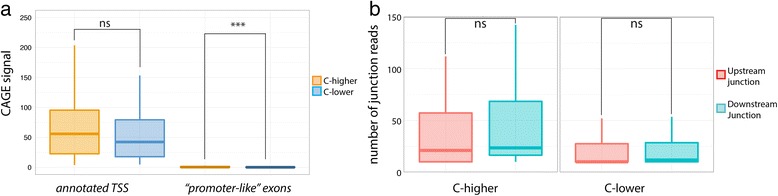
‘Promoter-like’ exons are not used as TSS. **a** Gene expression levels of exons in C-higher and C-lower conditions measured using CAGE tags. Distributions are given for ‘bona-fide’ annotated TSSs and for ‘promoter-like’ exons location. Significance levels are indicated by * (0.05 > *P* > 0.01), ** (0.01 > *P* > 0.001), *** (0.001 > *P*), and ns (*P* > 0.05). **b** Distribution of RNASeq junction reads in promoter like exons. Signals are represented for ‘promoter-like’ exons in C-higher and C-lower conditions. Significance levels are indicated by * (0.05 > *P* > 0.01), ** (0.01 > *P* > 0.001) and ‘ns’ (*P* > 0.05)

All these promoter associated features in ‘promoter-like’ exons could suggest that these exons actually overlap un-annotated TSS - in which case, elevated levels of H3K9ac, H3K27ac, and H3K4me3 could simply reflect the action of transcription, and be unrelated to exon inclusion. To rule out this possibility, we analyzed the number of CAGE tags (sequence tags that target specifically the 5’ end of transcripts [34]) mapping to ‘promoter-like’ exons, and find it marginal when compared to the level in annotated TSS (Fig. 6a). We also found no significant difference in the distribution of upstream and downstream junction inclusion reads (RNASeq reads that connect two neighboring exons), further confirming that ‘promoter-like exons’ are indeed ‘bona fide’ exons, and do not represent, as a bulk, un-annotated TSSs (Fig. 6b).

### R5. Proximity to the promoter and exon inclusion

**Fig. 7:**
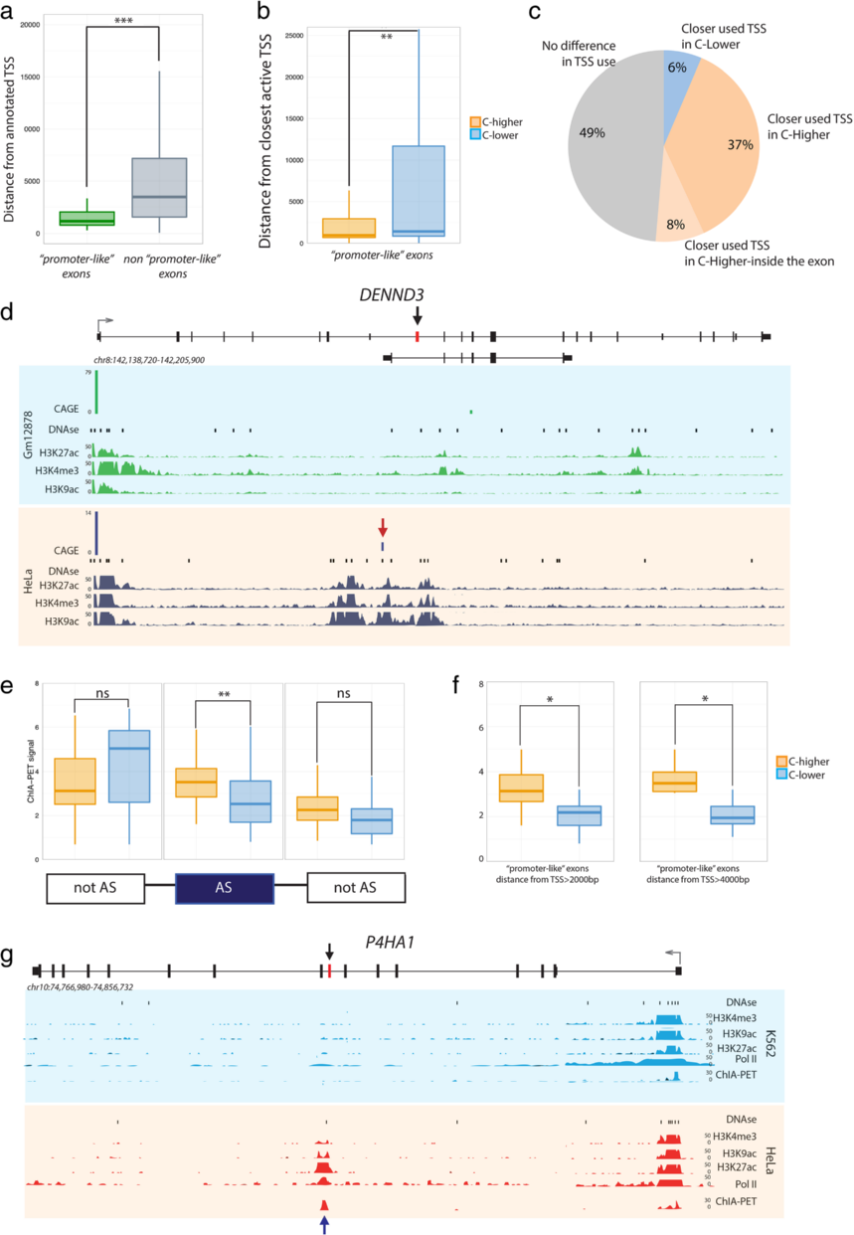
Relationship between promoter and ‘promoter-like’ exons. **a** Distribution of the distance (in nucleotides) between annotated TSS of ‘promoter-like’ and non-‘promoter-like’ exons. **b** Distribution of the distance (in nucleotides) between ‘promoter-like’ exons and the nearest active TSS in C-higher and C-lower cell lines. **c** Proportion of ‘promoter-like’ exons in which the active TSS is closer in C-higher than in C-lower cell lines, in C-lower than in C-higher cell lines, and at the same distance in C-higher and C-lower cell lines. The total number of exons displaying differences in TSS usage is 51. **d** USCS Genome browser view of the DENND3 gene, that contains a ‘promoter-like’ exon (in red) more included in Hela (0.91) than in Gm12878 cells (0.75). Genomic tracks for CAGE, DNase I, and ChIPSeq of H3K9ac, H3K27ac, andH3K4me3 levels are displayed. The CAGE signal corresponding to the alternative active promoter, used in Hela, is marked with a red arrow. **e** ChIA-PET signal in ‘promoter-like’ exons in C-higher and C-lower cell lines. Signals are represented for regulated (AS) and flanking non-regulated (notAS) exons. Significance levels are indicated by * (0.05 > *P* > 0.01), ** (0.01 > *P* > 0.001), *** (0.001 > *P*), and ns (*P* > 0.05). **f** ChIA-PET in ‘promoter-like’ exons separated in bins according to their distance to the TSS. Significance levels are indicated by * (0.05 > *P* > 0.01), ** (0.01 > *P* > 0.001), *** (0.001 > *P*), and ns (*P* > 0.05). **g** USCS Genome browser view of the P4HA1 gene, that contains an exon (in red) more included in Hela (0.34) than in K562 (0.21) cells. Genomic tracks for DNase I, ChIA-PET, and ChIPSeq of Pol II, H3K9ac, H3K27ac, and H3K4me3 are displayed. The ChIA-PET signal, specific of Hela cells, is marked with a blue arrow. **h** H3K4me3, H3K9ac, and H3K27ac levels on ‘promoter-like’ exons and at the corresponding closest active TSS. The fold-change between the ChIPSeq signal in C-higher and C-lower is significantly higher (*P* value <2.2e-7) in the exon than in the TSS for all histone modifications considered

In spite of not being promoters themselves, ‘promoter-like’ exons are significantly closer to the annotated TSSs when compared to the rest of differentially included exons (Fig. 7a), and many of them are second exons of the transcript, with their upstream exon beginning at the TSS itself. This explains the enrichment of DHS and Pol II signals in the non-regulated exons upstream of ‘promoter-like exons’ (Figs. 5c, d) - albeit these enrichments are not significantly different between C-higher and C-lower cell lines, in contrast to the enrichments in the ‘promoter-like’ exons. Moreover, analyzing clusters of CAGE tags, which are assumed to indicate annotated or un-annotated TSS, we observed alternative TSS usage for ‘promoter-like’ exons between cell lines (Fig. 7b). We found that in 45 of the 100 ‘promoter-like’ exon comparisons, the active TSS closest to the exon is closer in the C-higher cell line than in the C-lower cell line, while in only six exon comparisons is the other way around (Fig. 7b, c). For eight exon comparisons we found CAGE clusters within the exonic region, suggesting that these exons could indeed correspond to new, un-annotated TSSs. This could explain the significant CAGE enrichment occupancy in ‘promoter-like’ exons when comparing C-higher and C-lower cell lines (Fig. 6a). Figure 7d shows an example of an exon with higher inclusion level in Hela than in Gm1278 and with a closer active TSS being used only in the cell line of higher inclusion.

These results suggest that regulated enrichment of histone modifications and other promoter-associated features in ‘promoter-like’ exons could be the consequence of the physical proximity between these exons and real promoters. Transcription activating histone modifications would thus be ‘co-opted’ to participate also in the regulation of inclusion of ‘promoter-like’ exons. To further test this hypothesis, we analyzed genome-wide Chromatin Interaction Analysis with Paired-End-Tag sequencing data (ChIA-PET [35]) that has been used to map long-range chromatin interactions. It was recently shown that some internal exons loop and physically interact with promoter and enhancers, and for this reason display ‘promoter-like’ or ‘enhancer-like’ chromatin marks, and are enriched for co-transcriptional splicing [36]. These datasets are available only for Hela and K562 and therefore we could only analyze a fraction of the exon comparisons. In ‘promoter-like’ exons we found enriched ChIA-PET signal in C-higher than C-lower cell lines (Fig. 7e) in a fashion independent of the distance between the TSS and the exon itself (Fig. 7f). Since these ChIA-PET datasets target RNA polymerase II with un-phosphorylated ser2, found in the transcription pre-initiation complex and marking gene promoters [37], this increase of signal should indicate increase looping and interaction between ‘promoter-like’ exons and TSSs. As a control, we did not find ChIA-PET signal enrichment in the set of all regulated exons (Additional file 1: Figure S10). Figure 7g shows a ‘promoter-like’ exon more included in Hela than in K562, exhibiting local peaks of DNase I, RNA Pol II, H3K9ac, H3K27ac, and H3K4me3 in Hela, but not in K562. The exon also shows ChIA-PET tags only in Hela cells.

When analyzing individual ChIA-PET links, we found that 94 % of the ‘promoter-like’ exons have at least one interaction with the un-phosphorylated pol II, in the C-higher cell line. This percentage is 68 % in the C-lower cell line, considering only the cell lines where we have data available. Genomic connections detected through ChIA-PET are not necessarily distal. To assess whether linear proximity in the genome could also explain the promoter like features of this set of exons, we used chromatin segmentation tracks produced by the ENCODE project (chromHMM [38]). These genome segmentations represent broad epigenomic domains, reflecting specific combinations of histone modifications and other chromatin features. Although the resolution of the chromHMM segmentations is limited, we found that for about 40 % of the ‘promoter-like’ exon comparisons the exon is located in a chromatin state that extends all the way to the closest used TSS, both in the C-higher and C-lower cell lines, which would indicate linear proximity in the genome.

Linear and/or spatial proximity to active promoters explains the enrichment in histone modifications in included ‘promoter-like’ exons, but it does not necessarily show that there is a mechanistic connection between exon inclusion and levels of histone modifications. In fact, we found a positive correlation for these three histone marks between the levels at the TSS and at the exon, consistently higher and only significant (*P* value <0.01) in C-higher cell lines (Additional file 1: Table S10). However, while accumulation of the marks is generally higher in C-higher than C-lower cell lines, both at the TSS and at the exon, the difference in the levels of the histone modifications between C-higher and C-lower cell lines is much larger at the exons than at the TSS (*P* value <2.2e-7 (Fig. 7h). This strongly suggests that the enrichment of chromatin marks concomitant with inclusion in ‘promoter-like’ exons it is not a mere consequence of proximity to the promoter, but that it is mechanistically connected to the splicing of these exons.

## Discussion

Regulated alternative splicing is assumed to contribute to cell type identity and methods have been developed which are able to predict tissue specific exon inclusion with high accuracy [39]. In our analysis, we found a relatively small number of human exons (about 3 % of all exons) exhibiting regulated inclusion in a panel of human cell lines. This cannot be attributed to insufficient sampling by RNASeq, since ENCODE cell lines are sequenced in replicates at very high depth of coverage (around 240 M reads per sample). On the other hand, the diversity of biological samples used is certainly reduced, and cell lines are known to exhibit peculiar biology [40]. While regulated splicing, therefore, is likely to be more widespread than detected here, our results could also suggest that the contribution of splicing regulation to defining cell type identity is exerted chiefly through a relatively small, but well defined, set of exons.

Recent results have unveiled that pre-mRNA splicing occurs predominantly co-transcriptionally, thus providing a framework in which chromatin and transcription-related factors interact with the pre-mRNA processing machinery. However, among most exons with regulated inclusion we found in general, little direct association between differential inclusions and histone modifications. While these results are not fully unexpected, since splicing factors are likely to be the main players in splicing regulation, they somehow in contrast with reports of histone modifications influencing splicing outcomes through recruitment of splicing factors and through the modulation of RNA Pol II dynamics. Indeed, previous work linked high H3K9ac levels in the *NCAM* gene with fast elongating RNA Pol II and skipping of a specific exon [41]. High levels of H3K36me3 or H3K27me3 along the *FGFR2* were correlated with the regulation of a mutually exclusive alternative splicing event [10]. In these cases, however, changes in histone modifications appear to spread over large regions covering the whole gene, while here we explicitly explored chromatin modifications local to the exons. More importantly, in our work we investigated only direct effects acting independently, and we ignored the role of high order interactions between different histone modifications and other elements of chromatin structure. These could actually configure a quite complex histone-based splicing regulatory code. Furthermore, we focused specifically in complete exon skipping events, and ignored other types of splicing events such as alternative splice site usage. In this regard, Tilgner *et al.* [21] found that nucleosome occupancy may contribute more strongly to the definition to the 3’ splice site. If so, histone modifications would also be expected to play a major role in the regulation of alternative 3’ splice sites.

While we did not found evidence for a general direct effect of chromatin structure in exon inclusion, we did identify a subgroup of regulated exons (about 4 % of all regulated exons) for which co-occurrence of H3K9ac, H3K27ac, and H3K4me3, histone modifications typically associated to promoters, strongly correlate with exon usage levels. This association is not biased by our discovery approach, since we replicated it in cell line comparisons that were not part of the training set. These ‘promoter-like’ alternative exons appear predominantly in low abundance isoforms, but in which, a significant increase in the density of histone modification correlates with an increase in the levels of exon inclusion. These observations suggest that chromatin architecture may play a more prominent role in the regulation of exon inclusion, under conditions of weak splice site recognition.

We further related the accumulation of these histone modifications in highly included exons with higher occupancy of RNA Polymerase II. Accumulation of RNA Polymerase II has been linked to exon inclusion [42, 43], associated with slower Pol II kinetics and consequently additional opportunities for splice site recognition before competing sites come into play [44]. However we also found higher inclusion of ‘promoter-like’ exons in states of open chromatin, as measured by DNase I. This observation is somehow in contrast with previously proposed models linking closed chromatin and slower transcription elongation with increased exon inclusion [11, 45–47]. In particular for H3K9ac, previous studies reported a correlation between accumulation of this mark along the whole gene body (NCAM) and skipping of a specific alternative exon [47].

**Fig. 8:**
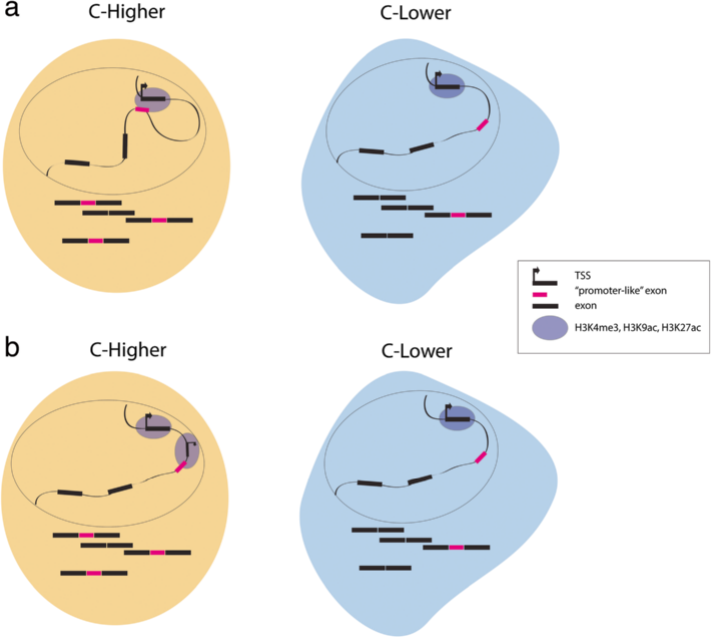
Models linking promoter activity with inclusion of ‘promoter-like’ exons. Looping model (**a**): physical interactions between TSS and the genomic region corresponding to the alternative exon facilitates exon inclusion (left), while absence of such interactions leads to more skipping of ‘promoter-like’ exons (pink). Alternative TSS model (**b**): the activation of an alternative TSS in the proximity of the genomic region corresponding to the alternative exon facilitates exon inclusion (left), while the inactivation of the TSS closer to the ‘promoter-like’ exon promotes its skipping

We also found that ‘promoter-like’ exons, while no promoters themselves, they are, on average, closer to TSS and enriched by ChIA-PET tags associated with RNA Polymerase II. Thus, we hypothesize that, in these exons, splicing regulation is mediated by the promoter, either by formation of a DNA loop with the exon and helping chromatin marking and transcription factor binding extending from the TSS to the alternative exon, or by differential splice site pairing when an alternative TSS generates an alternative first exon (Fig. 8). Recent work exploring the three-dimensional (3D) structure of the genome reported physical links between internal exons and their associated promoter or enhancers. These results argue for an interplay between 3D genome organization and alternative splicing regulation and warrant the systematic analysis of these associations in future studies using conformation capture technologies [48]. An alternative interpretation relies on the fact that H3K9ac, H3K27ac, H3K4me3, and DNase hypersensitive on ‘promoter-like’ exons simply reflect open chromatin on these exons. It is conceivable that binding of factors facilitated by the opening of chromatin influence splice site recognition either directly through their effects on splicing factor recruitment or through effects on RNA Pol II elongation - a mechanism resembling promotion of exon inclusion by CTCF [13].

## Conclusion

In summary, our work sheds light on functional connections between chromatin structure and pre-mRNA processing, establishing associations between epigenetic marks and differential exon inclusion and suggesting a role for promoter-like regions and 3D genome architecture in the regulation of the alternative splicing of certain exons. We specifically propose that in exons that are proximal to active promoter regions (either in linear or 3D space), open chromatin promotes exon inclusion, maybe by facilitating the recruitment of splicing factors. However, we want to stress that through our analysis we are unable to uncover the direction of the causation, and while histone modifications have been proposed to promote splicing, results have also been obtained suggesting that splicing can promote modification of histones by enhancing the recruitment of chromatin remodeling factors [49]. Further research will be needed to work out their detailed molecular mechanisms behind these observations.

## Methods

### Alternatively skipped exon calling

Using the gencode [50] v15 annotation we determined all exons that are

1. internal in all transcripts they appeared in
2. not overlapped by any non-identical exon
3. between 50 and 450 bps long
4. at least 600 nts away from the respective annotated TSS or TTS
5. surrounded by AG-GT splice sites
6. located on chromosomes 1–22 and X

For the remaining exons, a 2 × 2 table was constructed containing junction inclusion reads and junction exclusion reads in the two cell types (cell1 and cell2) retaining only the exons with a minimum of 1 junction inclusion read in cell1 and 1 exclusion read in cell2 or vice versa. For every cell pair, two one-sided Fisher tests were run and corrected for multiple testing in the Benjamini-Hochberg sense, resulting in three disjoint sets of exons:

1. exons that are significantly more included in cell1 (which will be referred to as ‘more included’, even though the choice of the direction from cell1 to cell2 is clearly arbitrary)
2. exons that are significantly less included in cell1 (which will be referred to as ‘less included’)
3. exons whose inclusion is not significantly changed between the two cell types (which will be referred to as ‘notAS exons’ for the sake of conciseness and clarity, although ‘non-significant AS exons’ would be more correct)

From the set of more and less included exons, we further selected the exons that met the following criteria:

i. the expression of the gene containing the exon did not change more than 10-fold between cell1 and cell2. To measure gene expression, we used CAGE tags mapping to the gene promoter (see below)
ii. at least 75 % of all positions in a 900 bp window around the acceptor were uniquely mappable for 36mers (see below)
iii. the inclusion levels of the exon changed by at least 0.1 or two-fold between the two cell lines

Genes frequently contained more than one alternatively spliced exon thus defined. In order to avoid gene specific bias that might be introduced by genes that contribute many alternative exons (as for example, the TTN gene where 212 exons passed the Fisher test), we chose only one upregulated and up to one downregulated exon per gene: The exon with the lowest *P* value among all exons for the gene in question.

For non-AS exons a similar procedure was carried out removing, however, the ‘inclusion changed by at least 0.1 or two-fold’ criterion and choosing the exon per gene whose estimated inclusion change was minimal among all non-AS exons of that gene (instead of the exon with the smallest *P* value). Additional file 1: Figure S1 illustrates this approach.

### Exon triplets

For each regulated exon and each cell type comparison, we defined two non-regulated exons: The closest up- and downstream exon that

- appeared in a transcript together with the alternative exon
- that showed Benjamini-Hochberg-corrected *P* value of 0.05 or greater.

### Inclusion level calculation

Inclusion level (IncLevel) is a measure defined to describe the splicing status of the exons. It is computed as a function of the reads arguing for the inclusion of the exon (JIR) and reads arguing for exclusion (JER). Formally it is

$$ IncLevel=\frac{0.5*JIR}{0.5*JIR+JER} $$

A value of 0 represents a totally excluded exon, while a value of 1 represents a totally included exon. IDR (Irreproducible Discovery Rate), a measure widely used within the ENCODE project to assess reproducibility between replicates [51], was applied at a level of 0.01 and only the exons passing this filtered were used in the remaining analysis.

### Splice site strength measure

For each exon we used maxEnt [52] in order to calculate an acceptor score and a donor score and represented the ‘exon strength’ by the sum of these two scores.

### Gene expression calculation

We employed ENCODE provided CAGE-clusters filtered by an Hidden Markov Model algorithm (TSS-HMM) to differentiate between 5’ capped termini of Pol II transcripts and recapping events, and scored according to number of constituent CAGE tags [25].

We associated CAGE-clusters to the closest TSS within a radius of 100 nucleotides. We computed the expression of a given gene as the sum of the scores of CAGE-clusters associated to all genes’ TSSs.

### Mappability calculation

Mappability for the hg19 genome was calculated using the GEM-mapper [53] for 36 bp and 75 bp reads. For each acceptor in the genome, mappability was calculated in the direction of transcription.

### Exon selection for validation experiments

Out of the exons that had cell type-specific H3K9ac peaks that co-occurred with high exon inclusion, we selected a total of 12 exons for validation by RT-PCR and ChIP.

### Cell culture, RNA extraction, and RT-PCR analysis

K562 and Hela cells were grown in Dulbecco’s modified Eagle’s medium (Gibco BRL) supplemented with 10 % of fetal bovine serum (FBS), penicillin, and streptomycin. Gm12878 cells were grown in RPMI (Gibco 1640), supplemented with 15%FBS (Gibco BRL), glycine (SEE), penicillin, and streptomycin.

Total RNA was isolated using Qiagen RNeasy mini kit and re-suspended in RNAse-free water (Ambion). DNA digestion was performed using RNase-free DNase (Promega). DNA-free total RNA (1 μg) was used for RT − PCR using SuperScript III reverse transcriptase (Invitrogen), random hexamers and oligo dT. 5 % of the reaction was used for real time PCR (Applied Biosystem) together with the primers (Additional file 1: Table S4) following the manufacturer’s instructions.

### Chromatin immunoprecipitation (ChIP)

Cells were plated at a density of 2 × 10^5^ cells in 75 cm^2^ flasks and after 48 h of culture, incubated with 1 % (vol/vol) formaldehyde in culture medium for 10 min at room temperature. Cells were then washed in cold phosphate-buffered saline (PBS), harvested, and lysed in a buffer containing 1 % SDS, 10 mM EDTA, and 50 mM Tris/HCl pH 8.1, and sonicated in 15 mL tubes with Bioruptor UCD-200 Diagenode (ultrasonic wave output power 250 W, 30” on-30” off, 4 × 10’) to yield chromatin sizes of 150–300 bp. A total of 100 μg of DNA/sample were used for immunoprecipitation with 5 μg of anti-H3K9ac rabbit (ab4441), anti-H3 rabbit (ab1791), or control rabbit IgGs (Sigma-Aldrich). Co-precipitated DNA was then analyzed by quantitative real time PCR performed with Sybr Green mix (Applied Biosystem) according to the manufacturer’s instructions. The antibody against total H3 was used for normalization as well as a control to exclude the possibility that the effects observed are caused by differences in nucleosome occupancy. The primers used are listed in the Additional file 1: Table S1.

### Exonic average signal (EAS)

We used ENCODE signal tracks [54] for a given histone mark (for example, H3K4me3). This signal track represents a sequencing-depth-normalized read density for each position on the genome.

- For each exon and cell-line we averaged this signal over all positions of the exon using bwtool [55], in order to get a single value representing the H3k4me3 signal on the exon in the cell type in question
- We proceeded the same way for all other histone marks, CTCF, Pol II, Input DNA, DNAse I, different transcription factors, and ChIA-PET

### Differential signal of EAS in two cell lines

- In a cell-type comparison we calculated for each exon the difference between the EAS in cell type I and in cell type II
- We then considered the distribution of these difference values of all exons being studied
- Wilcoxon signed-rank test, with Bonferroni correction for multiple testing, was used to assess the significance between the groups of exons

### Distribution of exonic average signal (EAS) for C-higher and C-lower cell-lines

- Using for all 100 exons the C-higher cell line, we computed a distribution of DNAse I-EAS for C-higher
- Using the C-lower cell line in the same way, we computed a second distribution for the C-lower cell line
- These two distributions are shown for DNAseI in Fig. 5c. Figure 5d shows the same for Pol2. Similarly in Additional file 1: Figure S8, we show the same for transcription factors with a significant difference between C-higher and C-lower. In Fig. 7e, we show the same for ChIA-PET (using only K562 and HelaS3, because these are the only cell lines, for which ChIA-PET is available). In Fig. 7h, we show the distributions for histone modification levels in the exons and their closest used TSS

### Genomic interactions

Besides the average signal from ChIA-PET experiments we also quantified the number of interactions found, per exon, with the un-phosphorylated ser2 polymerase II using the R package GenomicInteractions [56].

### Exon clustering

Alternative spliced exons differentially expressed between cell lines were partitioned according to their histone modifications and control levels using a k-means clustering approach. Unsupervised k-means clustering was applied to ‘higher included’ and ‘lower included’ exons separately using the differential signals of H3K9ac, H3K4me3, H3K27ac, CTCF, and control, the variables that came out as significant in Fig. 3. When defining the number of clusters required, we tried 2, 3, 4, and 5. K = 4 maximized the number of exons clustered with a clear signal for the histone modifications we were looking at. Exon comparisons falling in cluster 3 and 4 of ‘higher included’ exons and in cluster 4 of ‘lower included’ exons were merged in the group of ‘promoter-like exons’. Each exon comparison was thus composed by one exon, one cell line with higher inclusion level (C-higher) and one cell line with lower inclusion level (C-lower).

### Distance to closest annotated TSS

The distance of an exon to the annotated TSS was calculated by measuring the genomic distance from the first nucleotide of the exon and the closest TSS from all the transcripts the exon belongs to.

### Distance to closest used TSS

The distance of an exon to the closest used TSS was calculated by finding the closest CAGE-cluster from the TSS-HMM with a minimum expression value of 1 and calculating its genomic distance to the studied exon, in an annotation independent manner.

### Binding motifs analysis

A hyper geometric test, with Benjamin-Hochberg *P* value correction, was applied to JASPAR CORE 2014 [57] and MEME 4.4 [58] databases looking for enrichment in binding transcription factor and RNA binding proteins motifs inside the exons, respectively, in the ‘promoter-like exons’ versus the remaining differentially included identified exons.

### Splicing change in NHEK

NHEK, a cell line not used in the discovery analysis of the paper, was used for validation of our findings. The difference in inclusion and in histone modification signal was calculated between NHEK and K562, Gm12878, HepG2, HUVEC, and HeLaS3. Only exons with an inclusion difference larger than 0.1 and total chromatin difference larger than 1 were used. Total signal was calculated as the sum of H3K27ac, H3K4me3, and H3K9ac differences.

### Exon inclusion in GTEx project

We calculate inclusion levels, as described above, for the set of all internal exons in the 1,493 postmortem samples available from the GTEx project. The samples are very heterogeneous, coming from up to 43 different tissues from 175 individuals.

## Additional files

Additional file 1: **Supplementary Material.** (DOCX 21569 kb)

Additional file 2: **Set of differentially included exons selected in all cell pairs used, subset of more included exons.** This file is in bed format and field 4 is the cell pair in which the exon was identified as regulated. (BED 69 kb)

Additional file 3: **Set of differentially included exons selected in all cell pairs used, subset of less included exons.** This file is in bed format and field 4 is the cell pair in which the exon was identified as regulated. (BED 90 kb)

Additional file 4: **List of ‘promoter-like’, provided in bed format.** (BED 2 kb)

## Abbreviations

Bp: base pairs
CAGE: Cap analysis gene expression
ChIA-PET: Chromatin Interaction Analysis with Paired-End-Tag sequencing
ChIP: chromatin immunoprecipitation
ChIPSeq: sequencing of chromatin immunoprecipitation
CTCF: CCCTC-binding factor
DHS: DNase I hypersensitivity sites
DNA: Deoxyribonucleic acid
ENCODE: Encyclopedia of DNA Elements
GTEx: Genotype-Tissue Expression project
H3K27ac: H3 lysine 27 acetylation
H3K4me3: H3 lysine 3 trimethylation
H3K9ac: H3 lysine 9 acetylation
IDR: Irreproducible discovery rate
JER: Junction exclusion read
JIR: Junction inclusion read
mRNA: Messenger ribonucleic acid
NMD: Nonsense mediated decay
PCA: Principal component analysis
Pol: Polymerase
PolyA+: Polyadenylated
qPCR: quantitative real-time polymerase chain reaction
RNA: Ribonucleic acid
RNASeq: Sequencing of RNA
RT-PCR: Reverse transcription polymerase chain reaction
TF: Transcription factor
TSS: Transcription start site
TTS: Transcription termination site
