# Erratum to: Promoter-like epigenetic signatures in exons displaying cell type-specific splicing

**DOI:** 10.1186/s13059-016-0890-7

**Published:** 2016-03-18

**Authors:** Joao Curado, Camilla Iannone, Hagen Tilgner, Juan Valcárcel, Roderic Guigó

**Affiliations:** Centre for Genomic Regulation (CRG), The Barcelona Institute of Science and Technology, Dr. Aiguader, 88, Barcelona, 08003 Catalonia Spain; Graduate program in Areas of Basic and Applied Biology, Abel Salazar Biomedical Sciences Institute, University of Porto, Porto, 4099-003 Portugal; Universitat Pompeu Fabra, Dr. Aiguader, 88, Barcelona, 08003 Catalonia Spain; Department of Genetics, Stanford University, 300 Pasteur Dr., Stanford, 94305-5120 CA USA; Institució Catalana de Recerca i Estudis Avançats, Pg Lluis Companys 23, Barcelona, 08010 Catalonia Spain

After the publication of this work [1] an error was noticed in Fig. 7. The panel ‘h’ is missing from Fig. 7. Please see the corrected figure below. The publisher apologises for this error.

**Fig. 7:**
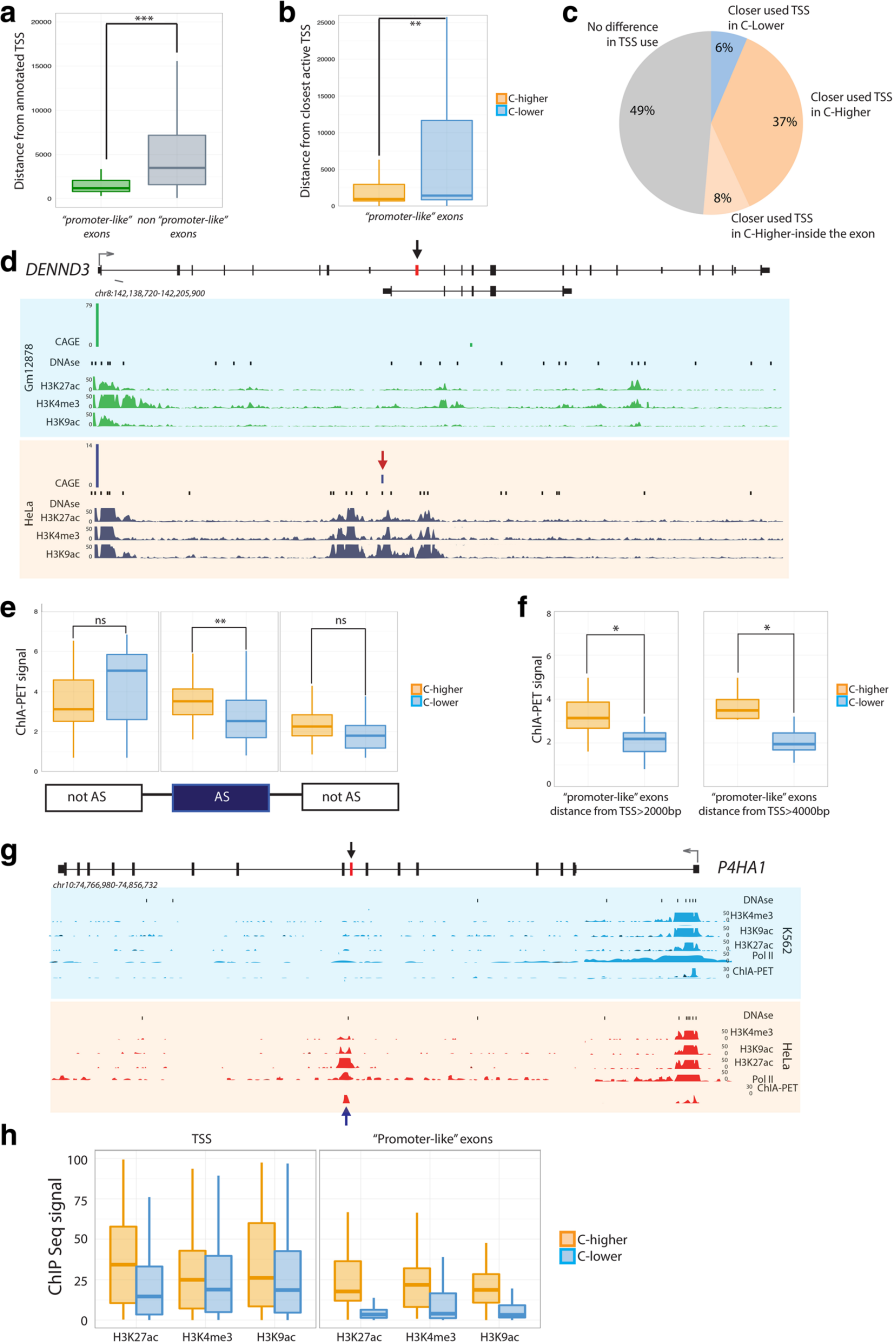
Relationship between promoter and ‘promoter-like’ exons. **a** Distribution of the distance (in nucleotides) between annotated TSS of ‘promoter-like’ and non-‘promoter-like’ exons. **b** Distribution of the distance (in nucleotides) between ‘promoter-like’ exons and the nearest active TSS in C-higher and C-lower cell lines. **c** Proportion of ‘promoter-like’ exons in which the active TSS is closer in C-higher than in C-lower cell lines, in C-lower than in C-higher cell lines, and at the same distance in C-higher and C-lower cell lines. The total number of exons displaying differences in TSS usage is 51. **d** USCS Genome browser view of the DENND3 gene, that contains a ‘promoter-like’ exon (in red) more included in Hela (0.91) than in Gm12878 cells (0.75). Genomic tracks for CAGE, DNase I, and ChIPSeq of H3K9ac, H3K27ac, andH3K4me3 levels are displayed. The CAGE signal corresponding to the alternative active promoter, used in Hela, is marked with a red arrow. **e** ChIA-PET signal in ‘promoter-like’ exons in C-higher and C-lower cell lines. Signals are represented for regulated (AS) and flanking non-regulated (notAS) exons. Significance levels are indicated by * (0.05 > *P* > 0.01), ** (0.01 > *P* > 0.001), *** (0.001 > *P*), and ns (*P* > 0.05). **f** ChIA-PET in ‘promoter-like’ exons separated in bins according to their distance to the TSS. Significance levels are indicated by * (0.05 > *P* > 0.01), ** (0.01 > *P* > 0.001), *** (0.001 > *P*), and ns (*P* > 0.05). **g** USCS Genome browser view of the P4HA1 gene, that contains an exon (in red) more included in Hela (0.34) than in K562 (0.21) cells. Genomic tracks for DNase I, ChIA-PET, and ChIPSeq of Pol II, H3K9ac, H3K27ac, and H3K4me3 are displayed. The ChIA-PET signal, specific of Hela cells, is marked with a blue arrow. **h** H3K4me3, H3K9ac, and H3K27ac levels on ‘promoter-like’ exons and at the corresponding closest active TSS. The fold-change between the ChIPSeq signal in C-higher and C-lower is significantly higher (*P* value <2.2e-7) in the exon than in the TSS for all histone modifications considered
