# Supplementary material for: Promoter-like epigenetic signatures in exons displaying cell type-specific splicing
Source: Genome Biol. 2015 Oct 23;16:236. doi: 10.1186/s13059-015-0797-8 (PMC4619081; doi:10.1186/s13059-015-0797-8)
Supplement: Supplementary file 1 — Supplementary Material. (DOCX 21569 kb) [file 13059_2015_797_MOESM1_ESM.docx]

- **Figure S1. Flowchart of the pipeline employed to define differentially included exons between two cell lines**
-
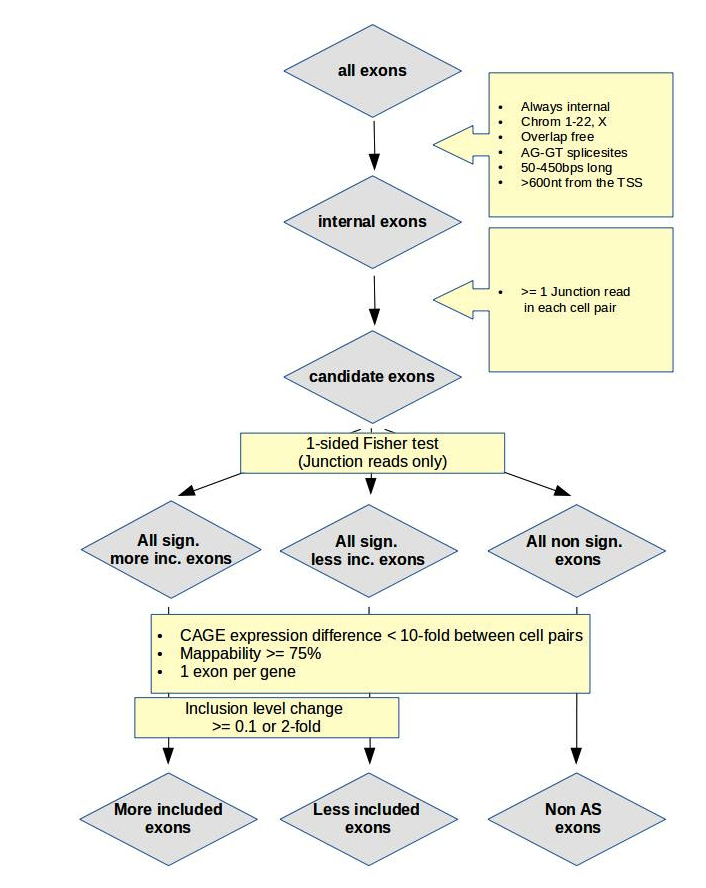


Internal exons were selected based on Gencode 15 annotation. Junction reads from RNASeq datasets of the cell types investigated within the ENCODE project were used to define the lists of alternative (more and less included) and not alternative (notAS) exons for each cell pair. These exons were also filtered by expression level changes (CAGE data), mappability, inclusion level change and only one per gene was kept.

**Figure S2. Differentially spliced exons in cell lines after filtering for gene expression change**

**
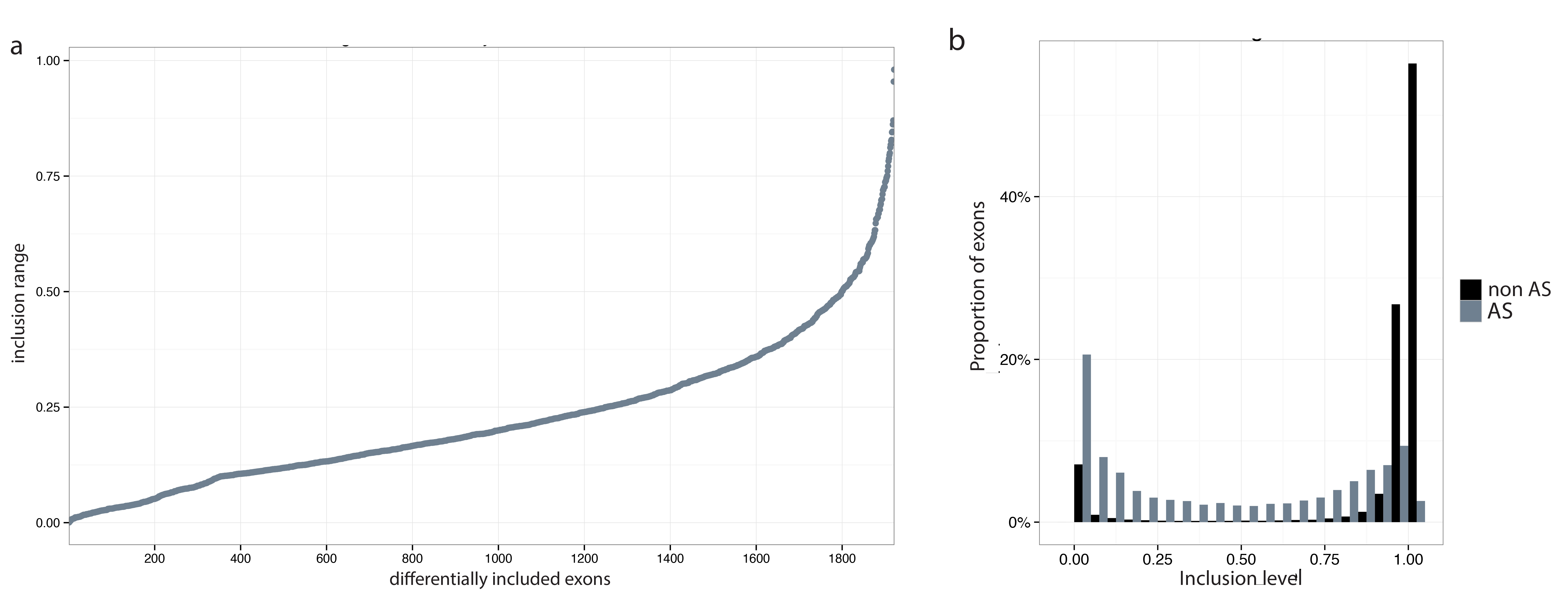
**

**
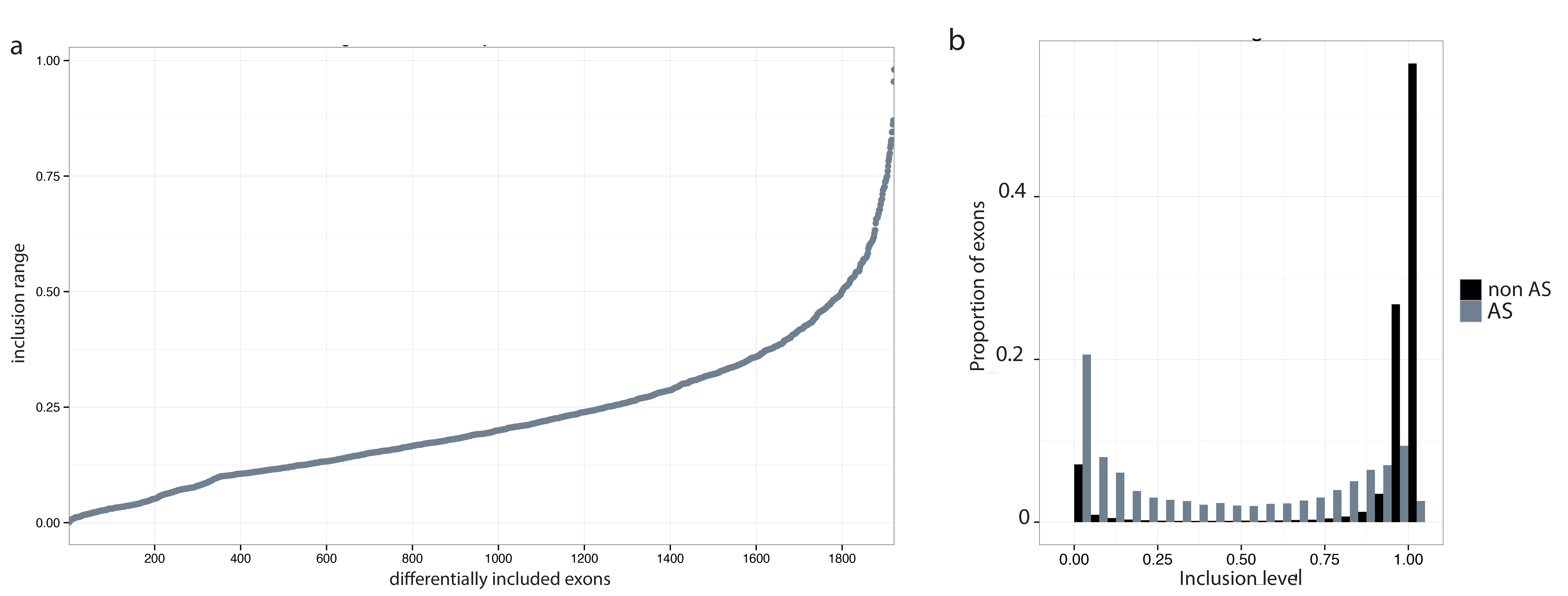
**

As in figure 2: **(a)** Inclusion range of regulated exons. The inclusion range of an exon is defined as the difference between the maximum and the minimum inclusion observed for that exon across the cell lines investigated. Exons are sorted by inclusion range. (**b)** Distribution of the inclusion level of regulated (AS) and non-regulated exons (notAS) across all the cell lines used.

**Figure S3. K-means clustering based on epigenetic signatures of non-regulated exons**

- **
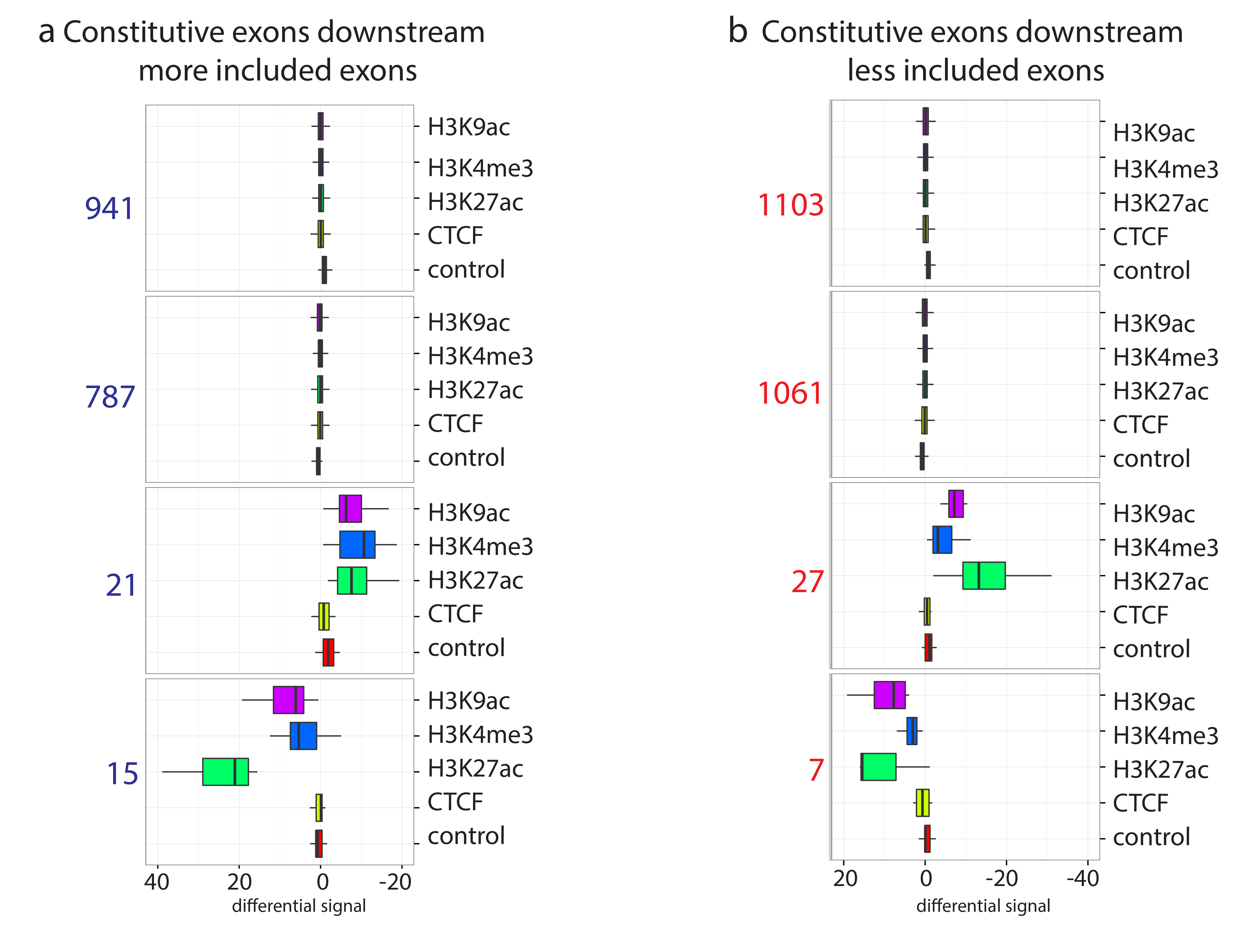
**
- We performed K-means clustering with non-regulated exons by using the first non regulated exon downstream of the regulated one using the exact same procedure as in Figure 4. In panel **(a)** boxplots represent differential ChIPSeq signal for the constitutive exons downstream of the more included exons and in panel **(b),** for the constitutive exons downstream of the less included ones. As opposed to Figure 4, we have clusters changing in both directions in each of the panels.
- **Figure S4. Principal component analysis (PCA) of the differentially included exons based on the differential H3K9ac, H3K27ac and H3K4me3 ChIPSeq signal.**
-
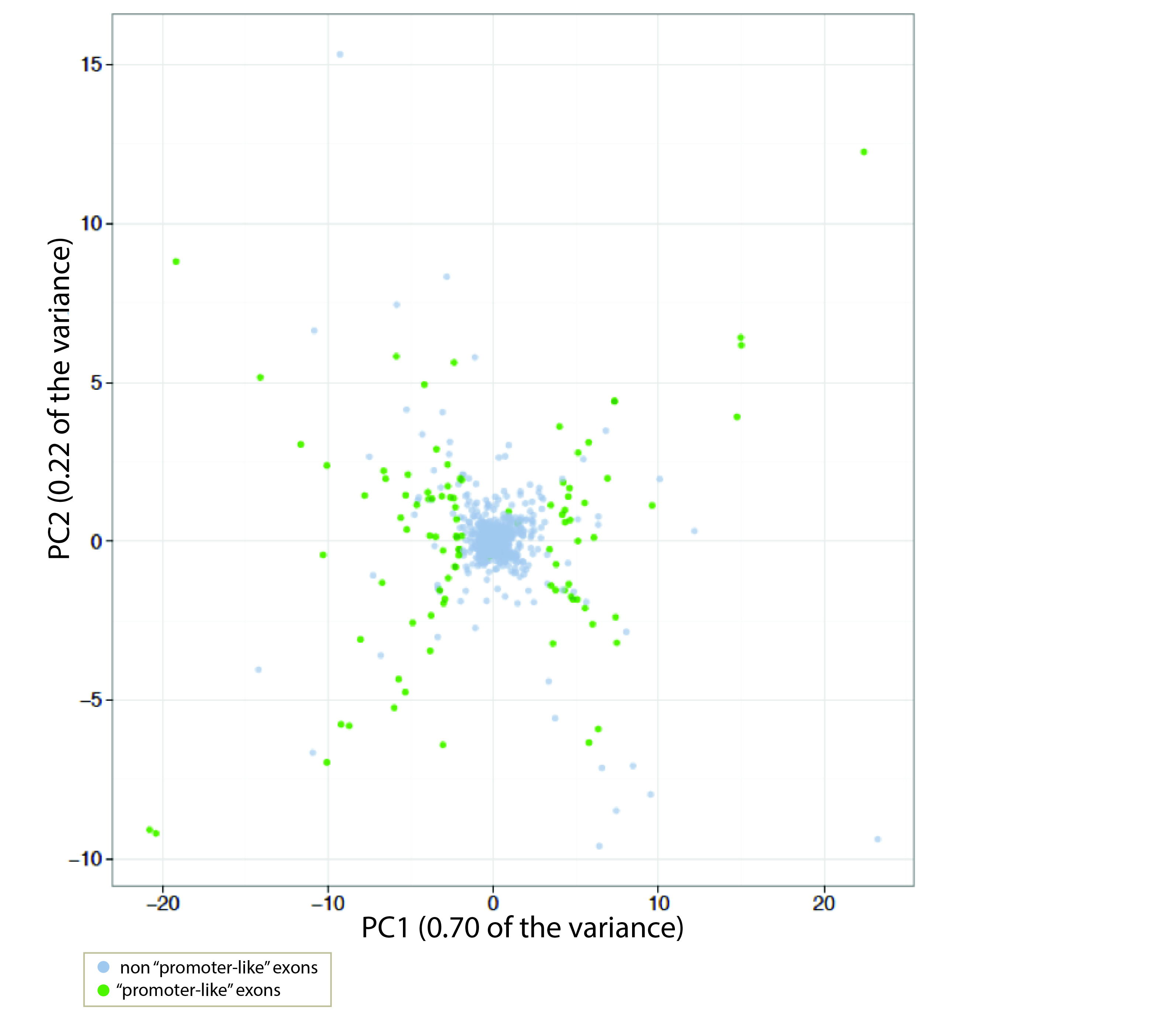

- “Promoter-like” exons are represented in green and the remaining differentially included exons in gray. The left and right subgroups of “promoter-like” exons, separated by PC1, represent the less and more included exons, respectively.
- **Figure S5. Differential inclusion and chromatin levels between K562 and NHEK**
-
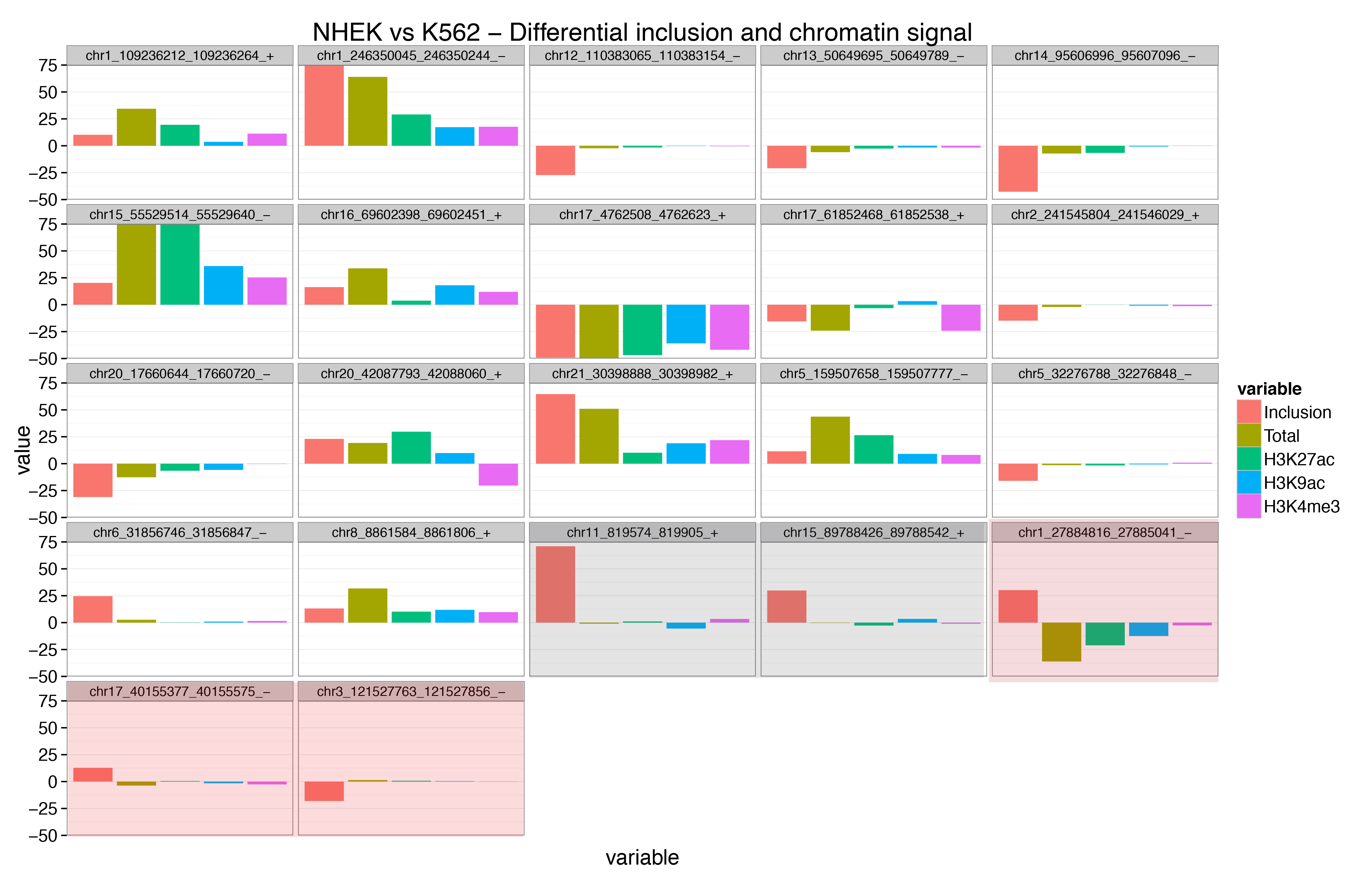

- In the 22 “promoter-like” exons that were regulated between K562 and NHEK we calculated the differential inclusion level (in the plot multiplied by 100), the differential signal of the H3K9ac, H3K27ac and H3K4me3 levels and the Total of the three. In the overwhelmingly majority of the cases (17), differential exon inclusion and differential total histone levels are in consistent directions. In three cases exon inclusion and histone modification levels change in opposite directions (pink background). In the remaining two cases, no change (Total <1) in histone modification levels could be detected (gray background).

**Figure S6** **Distribution of the inclusion level of “promoter-like” exons in C-higher or C-lower cell lines.**


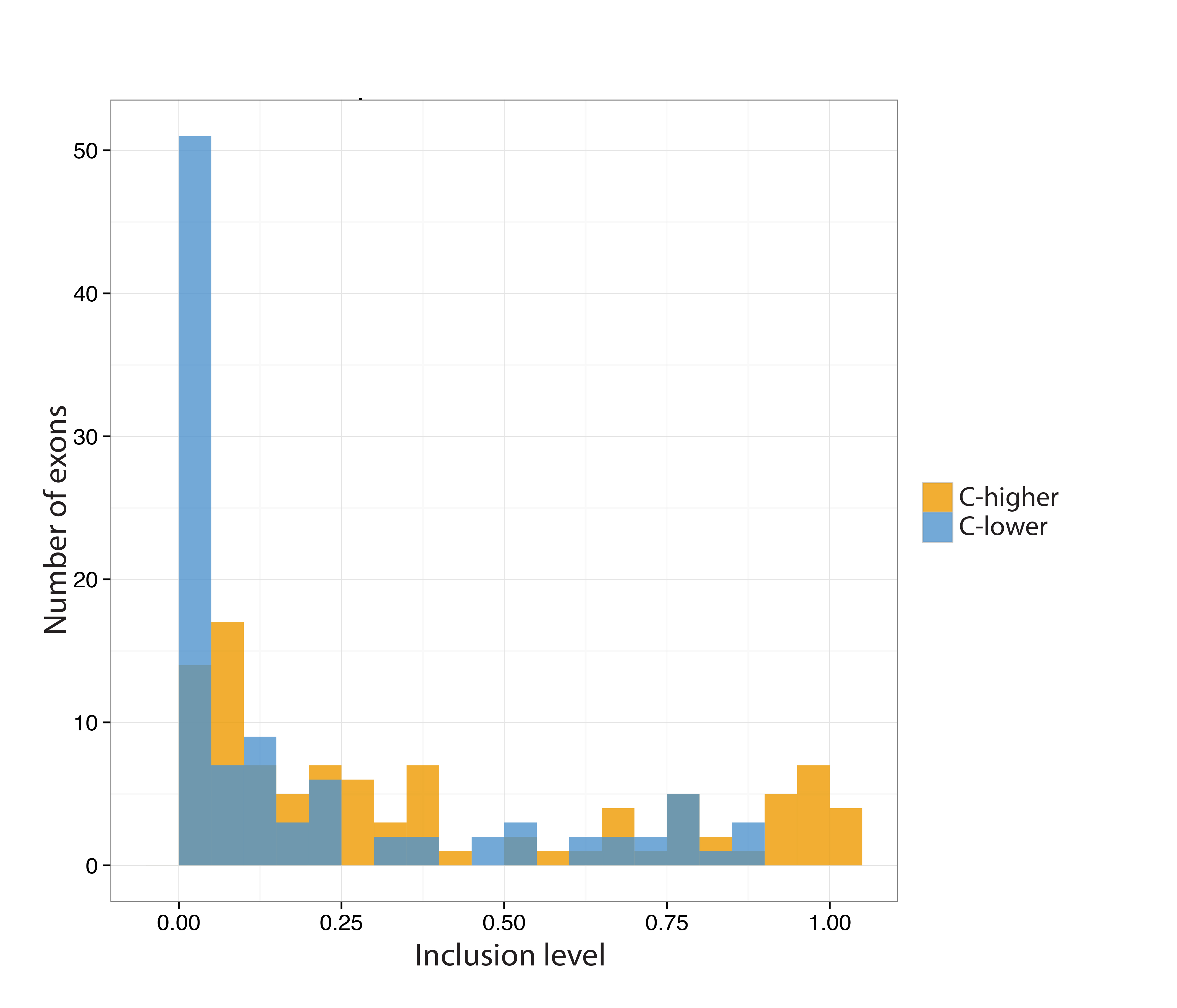


Inclusion levels for all “promoter-like” exons were calculated in C-higher and C-lower condition. There’s a shift towards higher inclusion levels in C-higher, as expected.

- **Figure S7. Inclusion levels distribution in tissue samples from GTEx.**


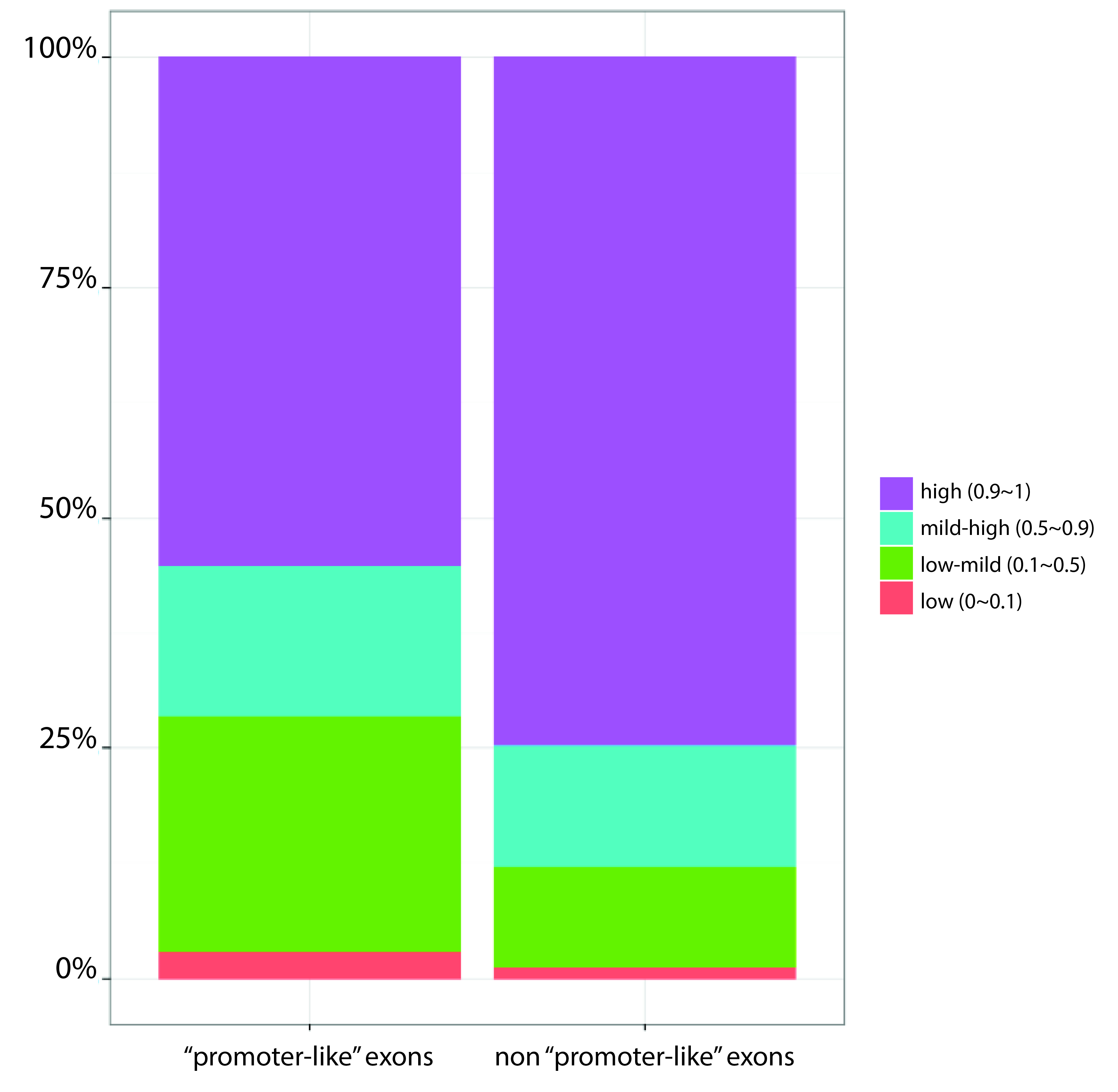


Inclusion levels for all the identified differentially included exons calculated in 1,493 RNASeq samples from the GTEx project. Each exon in each sample was categorized based on the inclusion level: low (between 0 ad 0.1), low-mid (between 0.1 and 0.5), mid-high (between 0.5 and 0.9) and high (between 0.9 and 1). Left plot represents “promoter-like” exons, right plot represents the remaining differentially included exons.

**Figure S8. Transcription Factors ChIPSeq signal in “promoter-like” exons in C-higher and C-lower condition.**

**
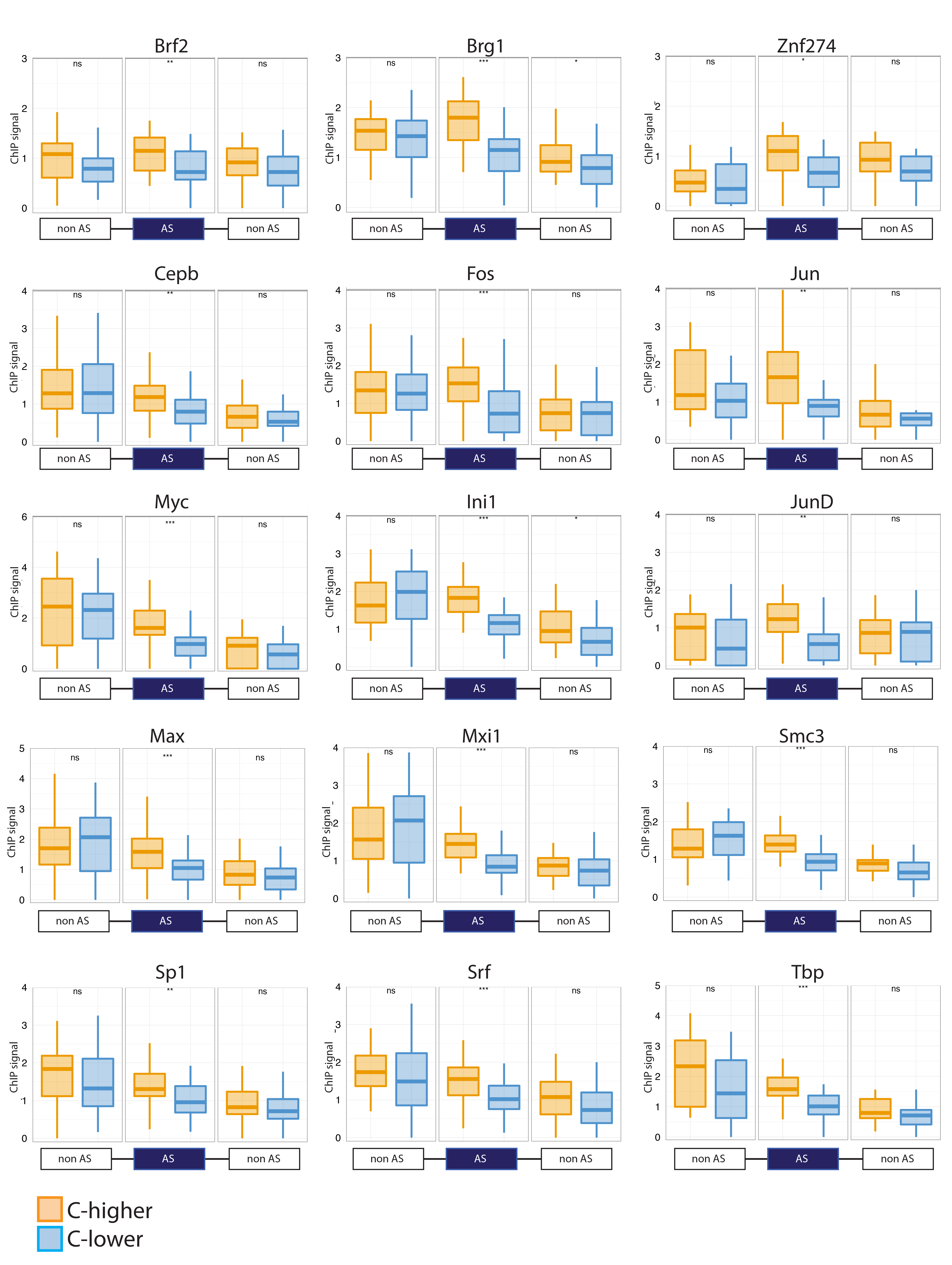
**

- Signals are represented for “promoter-like” and flanking non-differentially included exons. Significance levels are indicated by * (0.05>p>0.01), ** (0.01>p>0.001), *** (0.001>p) and “ns” (p>0.05). See figure S12 for input.

**Figure S9. Dnase I sensitivity signal in regulated exons.**

-
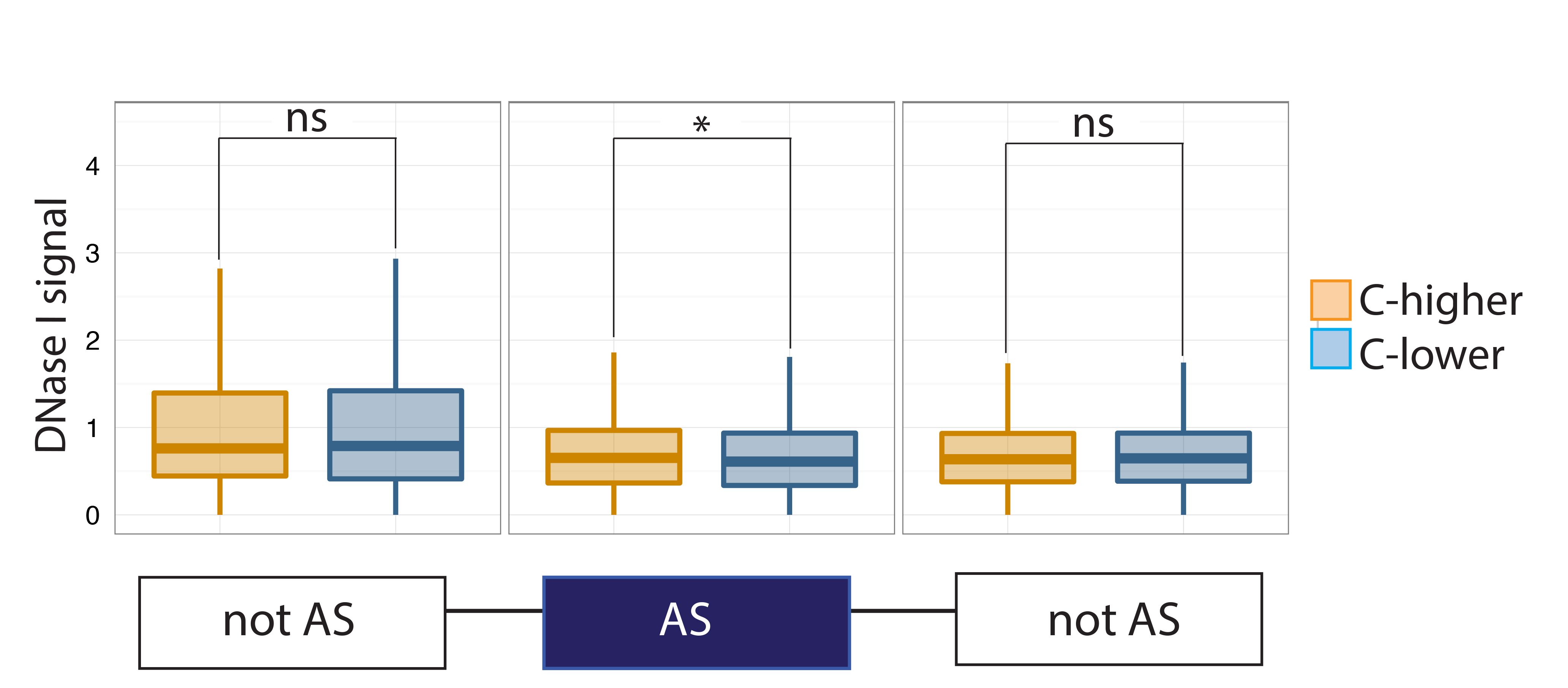

- Signals are represented for regulated and flanking non-regulated exons. Significance levels are indicated by * (0.05>p>0.01), ** (0.01>p>0.001), *** (0.001>p) and “ns” (p>0.05)

**Figure S10. ChIA-PET signal in regulated exons.**


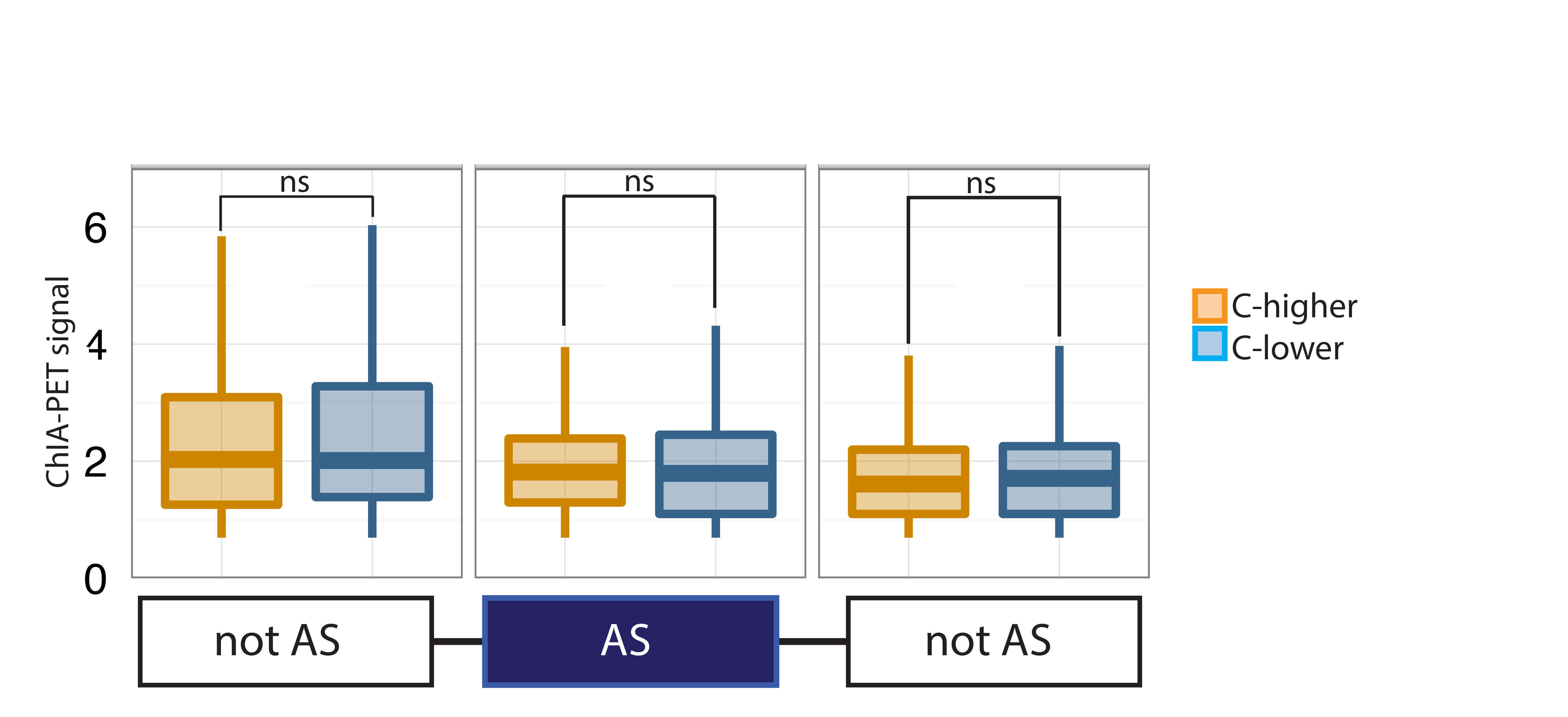


- ­­
- Signals are represented for regulated and flanking non-regulated exons. Significance levels are indicated by * (0.05>p>0.01), ** (0.01>p>0.001), *** (0.001>p) and “ns” (p>0.05)

**Figure S11. Input signal in “promoter-like” exons**


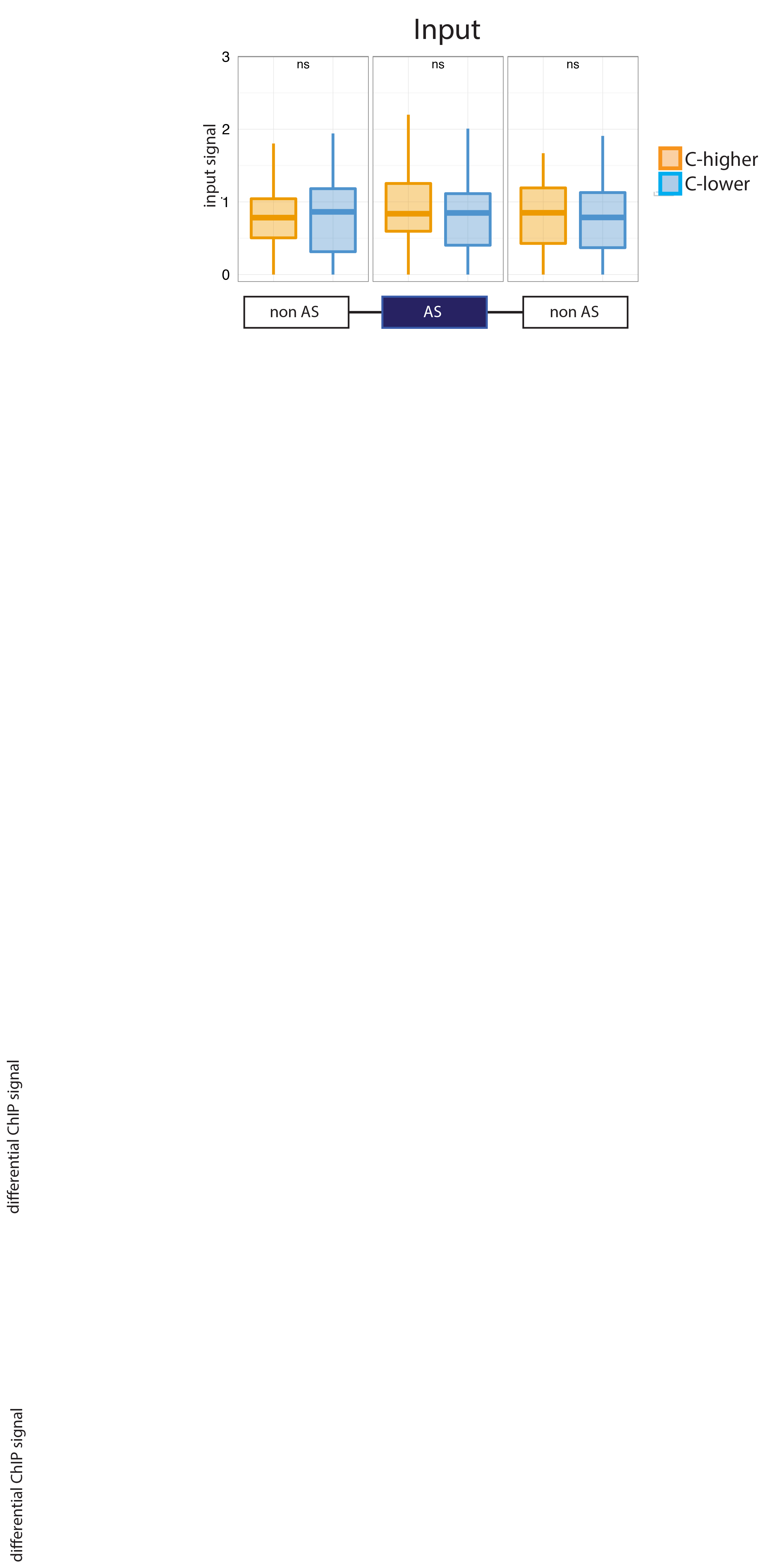


- Signals are represented for regulated and flanking non-regulated exons. Significance levels are indicated by * (0.05>p>0.01), ** (0.01>p>0.001), *** (0.001>p) and “ns” (p>0.05)
- **Table S1. List of primers used for exon inclusion validation**

| - **Primer name** | - **sequence** |
| --- | --- |
| - IFNGR1_dwr | - GACCTGTGGCATGATCTGGT |
| - IFNGR1_aef | - GCAGATGCTTTTGAAGTCACC |
| - IFNGR1_inclr | - AACATTAGTTGGTGTAGGCACTCC |
| - IFNGR1_skipr | - ACATTAGTTGGTGTAGGCACTGAG |
| - RNASET2_upf | - CAGCTGGCAGCGTTCTCT |
| - RNASET2_dwr | - GGCCAGTGCTGAACCATAAT |
| - RNASET2_skipf | - CAAGCGCCTGCGTGAC |
| - Camkk2_inclf | - TGCCGGAAATCAAGCTG |
| - Camkk2_inclr | - AGCGTGCAGTTCTCATCCTC |
| - Camkk2_skipf | - TGCCGGAAATCAAGATCC |
| - Camkk2_skipr | - GCTGACAGTGAGCGTTCCTC |
| - Camkk2_dwf | - TAAACGCTCCTTTGGGAACC |
| - Camkk2_dwr | - GCTGACAGTGAGCGTTCCTC |
| - LSM7_upf | - GGAGAAGAAGAAAAAGGAGAGC |
| - LSM7_upr | - TTTACCCGGATCGTCTTGTC |
| - LSM7_inclR | - GGCTAGGGCCTGTGACTCTTC |
| - LSM7_skiplR | - CAGGATTCCACTGGCTTCG |
| - LSM7_dwf | - GAATCCTGAAGGGCTTCGAC |
| - LSM7_dwr | - CTCGCATGTACTCAATGGTG |
| - LSM7_aef | - CCTCAGTGCTCACATCTCTCC |
| - LSM7_aer | - TGCAGATGTTCAGGTCCTTG |
| - rab27a_upf | - TGAAGAGGACATGTGATTGGA |
| - rab27a_upr | - ACAGGGTAGAGAACCGCTTG |
| - rab27a_aef | - TATCCACGGGCTAGCCATAC |
| - rab27a_aer | - TCCTCAGAGTGCTTCAGTGC |
| - rab27a_dwf | - TGGGAGACTCTGGTGTAGGG |
| - rab27a_dwr | - TCAATGCCCACTGTTGTGATA |
| - rab27a_inclr | - GGATAAGGGCAGAGCCTCTTTA |
| - rab27a_skipf | - CGGTTCTCTACCCTGTAAAGGTG |
| - MUYTH_inclr | - CCAGGGACCTGTATGGGTT |
| - MUYTH_inclf | - GCCAGGGACCTGTATGTAGA |
| - MUYTH_aef | - AAGTGATCTGCCCATCTTGG |
| - MUYTH_aer | - AGAACTGATAGCTCCCATGGAT |
| - MUYTH_dwf | - AAAAGGTCCCAGGTGTCCTC |
| - MUYTH_dwr | - CTGCACTGTTGAGGCTGTGT |
| - ABI1_inclf | - CAATTTTCTGCTCAGCCTCA |
| - ABI1_inclr | - GGGTGGAGCAATAGAAATTGA |
| - ABI1_skipf | - TTTTCTGCTCAGCCTCATGTT |
| - ABI1_skipr | - GGAGTTGGACTATCAGCAATTGA |
| - ABI1_dwf | - CCTCCACCAGATGACATTCC |
| - ABI1_dwr | - TGCAGCCTCCTCATCTTCAT |
| - ERI1_upf | - GCATGGAGGATCCACAGAGT |
| - ERI1_dwr | - AAGTCACTCGCACTGGAGGT |
| - ERI1_inclf | - ACGTTGTCAATCTCATCCTGAAAC |
| - ERI1_skipR | - ATTTACACTGTTGAGTTTCCTCGG |
| - USP16_upf | - ATGAGGGGATGCAGTTATGG |
| - USP16_dwr | - TGTCCGTTTCTTTCCCATGT |
| - USP16_inclf | - CTCTGTCGCCGTGGGATA |
| - USP16_aer | - GAGATCGAGGTGGGAGGAC |
| - USP16_skipf | - CTGTCGCCGTGGATTGTT |
| - thrap3_upf | - CAGCTGCGATCTCTGTGGTA |
| - thrap3_dwr | - CTGGATCCCAGACACTACCC |
| - thrap3_inclf | - TCTGTGGTAGGCCCAGTCAA |
| - thrap3_aer | - AAAACTGAGGCAGCTGGAGA |
| - thrap3_skipf | - TCTGTGGTAGGCCCAGAAGTG |
| - SRSF6_upf | - CGAGCGCGTGATCGTAGA |
| - SRSF6_upr | - GCGGCTTCCGTAGCTGTAG |
| - SRSF6_aef | - TTGTGTGACCCTTGCCCTAT |
| - SRSF6_aer | - TCTAATGGCAAAAGGCTGCT |
| - SRSF6_dwf | - AAATACGGACCACCTGTTCG |
| - SRSF6_dwr | - GCCAACTGCACCGACTAGA |
| - SRSF6_inclr | - GCCCCATTGGTCATGC |
| - SRSF6_skipr | - TCCACCTCCACCACTGC |
| - EZH2_9Af | - TGGGTATATATTGCCTGTTGGA |
| - EZH2_9Ar | - CTTCTGCAGGTGCCATTCA |
| - EZH2_9_10f | - AGTGTTACCAGCATTTGGAGG |
| - EZH2_10r | - ACGTTTTGGTGGGGTCTTTA |
| - EZH2_9_9Af | - GCGGAAGAACACAGAAACAG |
| - EZH2_9_9Ar | - TCCTAGGTAGGAGTGGCAAA |

- **Table S2. List of cell line comparison used**

| - **Cell-pair** | - **1** | - **2** | - **3** | - **4** | - **5** |
| --- | --- | --- | --- | --- | --- |
| - K562 vs Gm12878 | - YES | - YES | - YES | - YES | - YES |
| - K562 vs HelaS3 | - YES | - YES | - YES | - YES | - YES |
| - K562 vs Hepg2 | - YES | - YES | - NO | - YES | - YES |
| - K562 vs Huvec | - YES | - YES | - NO | - NO | - YES |
| - Gm12878 vs HelaS3 | - YES | - YES | - YES | - YES | - YES |
| - Gm12878 vs Hepg2 | - YES | - YES | - YES | - YES | - YES |
| - Gm12878 vs Huvec | - YES | - YES | - YES | - NO | - YES |
| - Huvec vs HelaS3 | - YES | - YES | - YES | - YES | - YES |
| - Huvec vs Hepg2 | - YES | - YES | - YES | - YES | - YES |
| - HelaS3 vs Hepg2 | - YES | - YES | - YES | - YES | - YES |

| - **Criteria for pairwise AS exons validation** | |
| --- | --- |
| - **1** | - AS exons should have weaker splice sites |
| - **2** | - AS exons should be smaller |
| - **3** | - Conding AS exons should be more often divisible by 3 |
| - **4** | - Both categories should have similar gene expression |
| - **5** | - Both categories should have no difference in mappability |

- **Table S3. Number of selected differential included exons**

| - **Cell-pair** | - **More included exons** | - **Less included exons** |
| --- | --- | --- |
| - K562 vs Gm12878 | - 283 | - 227 |
| - K562 vs HelaS3 | - 199 | - 334 |
| - Gm12878 vs HelaS3 | - 208 | - 427 |
| - Gm12878 vs Hepg2 | - 153 | - 296 |
| - Huvec vs HelaS3 | - 369 | - 413 |
| - Huvec vs Hepg2 | - 196 | - 229 |
| - HelaS3 vs Hepg2 | - 276 | - 272 |
| - TOTAL | - 1684 | - 2198 |

**Table S4. List of primers used for H3K9ac validation**

| - **tag** | - **sequence** |
| --- | --- |
| - ABI1_11f | - CTCCCCCTATGCCTCAGTT |
| - ABI1_11r | - CACGAAGCCTGTGAGAGGTA |
| - ABI1_7f | - CCCCCAACAGTTCCTAATGA |
| - ABI1_7r | - GACTTCCAAGCCTAGCAGGA |
| - EZH2_9Af | - TGGGTATATATTGCCTGTTGGA |
| - EZH2_9Ar | - CTTCTGCAGGTGCCATTCA |
| - EZH2_9f | - TTTCATGCAACACCCAACAC |
| - EZH2_9r | - GGTCCACAAGGTTTGTTGTCT |
| - camkk2_16f | - ACGCTGGTCGAAGTGACTG |
| - camkk2_16r | - CAAGCTGGGAATGTGTTTGA |
| - camkk2_12f | - GCTGACTTTGGTGTGAGCAA |
| - camkk2_12r | - AGAAGATCTTGCGGGTCTCA |
| - LSM7_aef | - CCTCAGTGCTCACATCTCTCC |
| - LSM7_aer | - TGCAGATGTTCAGGTCCTTG |
| - LSM7_upf | - GGAGAAGAAGAAAAAGGAGAGC |
| - LSM7_upr | - TTTACCCGGATCGTCTTGTC |

**Table S5: Number of “promoter-like” exons in each cell line comparison.**

| **Cell line comparison** | **Number of "promoter-like" exons** | **Number of "more included" exons** | **Number of "less included" exons** |
| --- | --- | --- | --- |
| **HeLaS3-Gm12878** | 9 | 6 | 3 |
| **HepG2-Gm12878** | 15 | 14 | 1 |
| **HepG2-HeLaS3** | 15 | 12 | 3 |
| **HeLaS3-Huvec** | 15 | 5 | 10 |
| **HepG2-Huvec** | 12 | 9 | 3 |
| **GM12878-K562** | 17 | 8 | 9 |
| **HelaS3-K562** | 17 | 4 | 13 |

**Table S6: Prediction of differential exon inclusion direction in “promoter-like” exons using differential signal of H3K4me3, H3K9ac and H3K27ac.**

|  | **K562** | **Gm12878** | **Helas3** | **Huvec** | **Hepg2** |  |
| --- | --- | --- | --- | --- | --- | --- |
| **NHEK** | 17 | 14 | 15 | 12 | 15 | **Correct** |
|  | 3 | 3 | 6 | 7 | 8 | **Wrong** |

**Table S7: Clustered exons in each cell line comparison and their correspondent “C-higher” and “C-lower” cell line**

|  | **Exon** | **C-Higher** | **C-Lower** |
| --- | --- | --- | --- |
| HeLaS3-Gm12878 | chr15 89788426 89788542 + | Helas3 | Gm12878 |
|  | chr16 2965409 2965477 + | Helas3 | Gm12878 |
|  | chr16 87985787 87985879 + | Helas3 | Gm12878 |
|  | chr17 61852468 61852538 + | Helas3 | Gm12878 |
|  | chr20 42087793 42088060 + | Helas3 | Gm12878 |
|  | chr3 127310733 127310793 - | Helas3 | Gm12878 |
|  | chr8 142170731 142170886 + | Helas3 | Gm12878 |
|  | chr12 125346926 125347043 - | Gm12878 | Helas3 |
|  | chr2 85806132 85806290 + | Gm12878 | Helas3 |
| **HepG2-Gm12878** | chr12 110383065 110383154 - | Hepg2 | Gm12878 |
|  | chr1 29493241 29493424 - | Hepg2 | Gm12878 |
|  | chr1 39844133 39844195 + | Hepg2 | Gm12878 |
|  | chr16 23606750 23606959 - | Hepg2 | Gm12878 |
|  | chr17 40155377 40155575 - | Hepg2 | Gm12878 |
|  | chr17 61852468 61852538 + | Hepg2 | Gm12878 |
|  | chr19 12404047 12404190 - | Hepg2 | Gm12878 |
|  | chr20 47898289 47898355 + | Hepg2 | Gm12878 |
|  | chr2 177134944 177135147 + | Hepg2 | Gm12878 |
|  | chr2 178196870 178196979 - | Hepg2 | Gm12878 |
|  | chr2 216975190 216975274 + | Hepg2 | Gm12878 |
|  | chr2 241545804 241546029 + | Hepg2 | Gm12878 |
|  | chr6 56607069 56607203 - | Hepg2 | Gm12878 |
|  | chr7 102716224 102716328 + | Hepg2 | Gm12878 |
|  | chr8 129060694 129060855 + | Gm12878 | Hepg2 |
| **HepG2-HeLaS3** | chr16 23606750 23606959 - | Hepg2 | Helas3 |
|  | chr16 69602398 69602451 + | Hepg2 | Helas3 |
|  | chr16 84172828 84172911 - | Hepg2 | Helas3 |
|  | chr19 12404047 12404190 - | Hepg2 | Helas3 |
|  | chr20 47898289 47898355 + | Hepg2 | Helas3 |
|  | chr2 177134944 177135147 + | Hepg2 | Helas3 |
|  | chr2 216975190 216975274 + | Hepg2 | Helas3 |
|  | chr2 237995375 237995439 + | Hepg2 | Helas3 |
|  | chr2 73462678 73462773 + | Hepg2 | Helas3 |
|  | chr3 124450353 124450451 + | Hepg2 | Helas3 |
|  | chr7 143579016 143579192 - | Hepg2 | Helas3 |
|  | chr7 38218692 38218747 + | Hepg2 | Helas3 |
|  | chr16 20922505 20922586 + | Helas3 | Hepg2 |
|  | chr20 60926044 60926313 - | Helas3 | Hepg2 |
|  | chrX 71840575 71840751 - | Helas3 | Hepg2 |
| **HeLaS3-HUVEC** | chr1 27884816 27885041 - | Helas3 | Huvec |
|  | chr15 89788426 89788542 + | Helas3 | Huvec |
|  | chr16 2965409 2965477 + | Helas3 | Huvec |
|  | chr16 89753997 89754223 + | Helas3 | Huvec |
|  | chr2 10548688 10548860 + | Helas3 | Huvec |
|  | chr11 819574 819905 + | Huvec | Helas3 |
|  | chr12 110383065 110383154 - | Huvec | Helas3 |
|  | chr1 32539378 32539473 + | Huvec | Helas3 |
|  | chr17 4762508 4762623 + | Huvec | Helas3 |
|  | chr2 164591413 164591582 - | Huvec | Helas3 |
|  | chr2 237995375 237995439 + | Huvec | Helas3 |
|  | chr5 32276788 32276848 - | Huvec | Helas3 |
|  | chr6 44189323 44189422 + | Huvec | Helas3 |
|  | chr8 129060694 129060855 + | Huvec | Helas3 |
|  | chr9 125016553 125016646 - | Huvec | Helas3 |
| **HepG2-Huvec** | chr10 13150138 13150289 + | Hepg2 | Huvec |
|  | chr16 69602398 69602451 + | Hepg2 | Huvec |
|  | chr16 84172828 84172911 - | Hepg2 | Huvec |
|  | chr20 47898289 47898355 + | Hepg2 | Huvec |
|  | chr2 177134944 177135147 + | Hepg2 | Huvec |
|  | chr4 76440643 76440742 + | Hepg2 | Huvec |
|  | chr6 56607069 56607203 - | Hepg2 | Huvec |
|  | chr8 19315041 19315327 - | Hepg2 | Huvec |
|  | chr9 134379576 134379727 + | Hepg2 | Huvec |
|  | chr1 109236212 109236264 + | Huvec | Hepg2 |
|  | chr17 4762508 4762623 + | Huvec | Hepg2 |
|  | chr8 129060694 129060855 + | Huvec | Hepg2 |
| **GM12878-K562** | chr1 246116533 246116657 - | Gm12878 | K562 |
|  | chr13 50649695 50649789 - | Gm12878 | K562 |
|  | chr13 99960937 99961063 + | Gm12878 | K562 |
|  | chr14 95606996 95607096 - | Gm12878 | K562 |
|  | chr3 121527763 121527856 - | Gm12878 | K562 |
|  | chr6 137536404 137536514 - | Gm12878 | K562 |
|  | chr6 167368983 167369115 - | Gm12878 | K562 |
|  | chr6 30657053 30657229 - | Gm12878 | K562 |
|  | chr12 120109300 120109473 + | K562 | Gm12878 |
|  | chr1 246350045 246350244 - | K562 | Gm12878 |
|  | chr1 36691013 36691064 + | K562 | Gm12878 |
|  | chr15 55529514 55529640 - | K562 | Gm12878 |
|  | chr20 42087793 42088060 + | K562 | Gm12878 |
|  | chr21 30398888 30398982 + | K562 | Gm12878 |
|  | chr6 31856746 31856847 - | K562 | Gm12878 |
|  | chr8 129060694 129060855 + | K562 | Gm12878 |
|  | chr8 8861584 8861806 + | K562 | Gm12878 |
| **HelaS3-K562** | chr10 74804739 74804809 - | Helas3 | K562 |
|  | chr16 87985787 87985879 + | Helas3 | K562 |
|  | chr20 17660644 17660720 - | Helas3 | K562 |
|  | chr7 44789414 44789579 + | Helas3 | K562 |
|  | chr1 163292657 163292885 + | K562 | Helas3 |
|  | chr1 36691013 36691064 + | K562 | Helas3 |
|  | chr15 55529514 55529640 - | K562 | Helas3 |
|  | chr16 69602398 69602451 + | K562 | Helas3 |
|  | chr16 84172828 84172911 - | K562 | Helas3 |
|  | chr21 30398888 30398982 + | K562 | Helas3 |
|  | chr2 85806132 85806290 + | K562 | Helas3 |
|  | chr4 44682458 44682569 + | K562 | Helas3 |
|  | chr5 159507658 159507777 - | K562 | Helas3 |
|  | chr6 3002284 3002520 + | K562 | Helas3 |
|  | chr7 38218692 38218747 + | K562 | Helas3 |
|  | chr8 129060694 129060855 + | K562 | Helas3 |
|  | chr8 8861584 8861806 + | K562 | Helas3 |

- **Table S8. List of RBP binding sites enriched in “promoter-like” exons**

| - **ID** | - **RBP name** | - **organism** | - **db** | - **p-value** |
| --- | --- | --- | --- | --- |
| - RNCMPT00003 | - aret | - Drosophila_melanogaster | - RNAcompete | - 5.84E-18 |
| - RNCMPT00004 | - BRUNOL4 | - Homo_sapiens | - RNAcompete | - 8.73E-17 |
| - RNCMPT00011 | - papi | - Drosophila_melanogaster | - RNAcompete | - 7.60E-15 |
| - RNCMPT00027 | - HNRNPL | - Homo_sapiens | - RNAcompete | - 2.15E-10 |
| - RNCMPT00051 | - RBM38 | - Homo_sapiens | - RNAcompete | - 2.15E-10 |
| - RNCMPT00069 | - sm | - Drosophila_melanogaster | - RNAcompete | - 2.15E-10 |
| - RNCMPT00113 | - RBM4 | - Homo_sapiens | - RNAcompete | - 2.15E-10 |
| - RNCMPT00114 | - aret | - Drosophila_melanogaster | - RNAcompete | - 2.15E-10 |
| - RNCMPT00134 | - SRSF4 | - Homo_sapiens | - RNAcompete | - 2.15E-10 |
| - RNCMPT00150 | - ESRP2 | - Homo_sapiens | - RNAcompete | - 2.15E-10 |
| - RNCMPT00160 | - HNRNPH2 | - Homo_sapiens | - RNAcompete | - 2.15E-10 |
| - RNCMPT00166 | - CELF3 | - Homo_sapiens | - RNAcompete | - 2.15E-10 |
| - RNCMPT00178 | - hnRNPLL | - Homo_sapiens | - RNAcompete | - 6.66E-04 |
| - RNCMPT00179 | - Rbm24 | - Mus_musculus | - RNAcompete | - 8.08E-04 |
| - RNCMPT00216 | - RRM_1 | - Trypanosoma_brucei | - RNAcompete | - 1.01E-03 |
| - RNCMPT00246 | - Pcbp4 | - Mus_musculus | - RNAcompete | - 3.33E-03 |
| - RNCMPT00270 | - aret_CONSTRUCT | - RNAcompete_CONSTRUCTS | - RNAcompete | - 2.89E-02 |
| - RNCMPT00283 | - Rbm24 | - Mus_musculus | - RNAcompete | - 4.20E-02 |
| - RNCMPT00285 | - Rbm24 | - Mus_musculus | - RNAcompete | - 4.20E-02 |

- **Table S9. List of Transcription factor binding sites enriched in “promoter-like” exons**

| - **ID** | - **TF name** | - **organism** | - **db** | - **p-value** |
| --- | --- | --- | --- | --- |
| - MA0472.1 | - EGR2 | - Mus_musculus | - JASPAR core | - 0.00E+00 |
| - MA0592.1 | - ESRRA | - Homo_sapiens | - JASPAR core | - 3.69E-04 |
| - MA0599.1 | - KLF5 | - Homo_sapiens | - JASPAR core | - 6.15E-03 |
| - MA0491.1 | - JUND | - Homo_sapiens | - JASPAR core | - 1.23E-02 |
| - MA0477.1 | - FOSL1 | - Homo_sapiens | - JASPAR core | - 2.82E-02 |
| - MA0131.1 | - HINFP | - Homo_sapiens | - JASPAR core | - 3.74E-02 |
| - MA0470.1 | - E2F4 | - Homo_sapiens | - JASPAR core | - 3.74E-02 |

**Table S10: Correlation between average histone modification signal in the exon region and in the closest used TSS**

|  | **H3k9ac** | **H3k27ac** | **H3k4me3** |
| --- | --- | --- | --- |
| C-higher | 0.29* | 0.26* | 0.45* |
| C-lower | 0.22* | 0.05 | 0.19 |

- * indicates p-value < 0.01
